# PAX4 R192H variant impairs β cell function by disrupting β cell identity and compensatory capacity in response to metabolic stress

**DOI:** 10.1186/s13073-026-01669-z

**Published:** 2026-05-28

**Authors:** Jungsun Park, Kyun Hoo Kim, Hyunsuk Lee, Joon Ho Moon, Soo Heon Kwak, Jong Il Kim, Hail Kim, Kyong Soo Park

**Affiliations:** 1 Graduate School of Medical Science and Engineering, Korea Advanced Institute of Science and Technology, 193 Munji-Ro, Yuseong-Gu, KAIST, Daejeon, 34051 Republic of Korea; 2 Department of Biochemistry and Cell Biology, School of Medicine, Kyungpook National University, Daegu, Korea; 3 Department of Translational Medicine, Seoul National University College of Medicine, Seoul, Korea; 4 Department of Internal Medicine, Seoul National University Hospital, Seoul National University College of Medicine, Seoul, Korea; 5 Medical Research Center, Genomic Medicine Institute, Seoul National University College of Medicine, Seoul, Korea; 6 Department of Internal Medicine, Seoul National University Bundang Hospital, Seoul National University College of Medicine, 82 Gumi-ro, 173 Beon-Gil, Gumi-Ro, Bundang-Gu, Seongnam-Si, Gyeonggi-Do 13620 Republic of Korea; 7 Department of Biomedical Sciences, Seoul National University College of Medicine, 103, Daehak-Ro, Jongno-Gu, Seoul, 03080 Republic of Korea; 8 Biomedical Research Center, Korea Advanced Institute of Science and Technology, Daejeon, Korea; 9 Department of Internal Medicine, Konkuk University Medical Center, 120-1, Neungdong-Ro, Gwangjin-Gu, Seoul, 05030 Republic of Korea

**Keywords:** PAX4, Type 2 diabetes genetics, Pancreatic β-cell

## Abstract

**Background:**

Type 2 diabetes is characterized by progressive β cell dysfunction, yet the mechanisms by which genetic susceptibility contributes to β cell area and function remain poorly understood. PAX4 is a transcription factor critical for β cell development, and a nonsynonymous variant resulting in an arginine-to-histidine substitution at position 192 (R192H) has been associated with increased type 2 diabetes risk and identified only in individuals of East Asian ancestry.

**Methods:**

Here, we generated *PAX4* R192H knock-in (*Pax4*^R192H/R192H^) mouse and integrated metabolic phenotyping, bulk and single cell transcriptomics, and human cohort analyses to investigate how *PAX4* R192H mutation increases the risk of type 2 diabetes.

**Results:**

Homozygote knock-in mice (*Pax4* R192H) exhibited normal pancreatic endocrine development but developed glucose intolerance and impaired insulin secretion when fed a high-fat diet. Bulk and single-cell RNA-seq of islets from *Pax4* R192H mice fed high-fat diet revealed impaired β cell adaptation to metabolic stress characterized by enhanced endoplasmic reticulum stress and impaired β cell maturity, with upregulation of dedifferentiation and α cell markers and downregulation of β cell identity genes. *Pax4* deletion in β cells resulted in similar phenotypic and transcriptomic profiles to *Pax4* R192H mice. In humans, the trajectories of β cell function were evaluated over a 14-year period using biennial oral glucose tolerance tests from 4,242 participants, where *PAX4* R192H carriers showed 1.4-fold accelerated decline in disposition index, with increasing body mass index further exacerbating their type 2 diabetes risk.

**Conclusions:**

Overall, PAX4 is essential for maintaining β cell identity and compensatory function under metabolic stress, and the R192H variant predisposes to type 2 diabetes by impairing this adaptive capacity.

**Supplementary Information:**

The online version contains supplementary material available at 10.1186/s13073-026-01669-z.

## Background

Type 2 diabetes is a major public health challenge, currently affecting over 500 million people [1]. Genome-wide association studies have identified genetic variants associated with type 2 diabetes across diverse populations, highlighting the heterogeneous nature of its pathogenesis [2–4]. Additionally, our previous study using whole-exome sequencing and exome array genotyping identified a nonsynonymous variant in the paired box 4 (*PAX4*) gene, rs2233580 (chr7:127,613,496 C > T; p.Arg192His, hereafter referred to as *PAX4* R192H), as a type 2 diabetes-associated allele with specificity to East Asian populations. This variant is associated with increased type 2 diabetes risk (~ 1.8-fold for heterozygote and 3.2-fold for compound homozygote) and an earlier age of disease onset in Koreans [5].

PAX4 is a transcription factor that is essential for pancreatic β cell development; *Pax4* knockout (KO) mice fail to generate β cells [6]. While the role of PAX4 in β cell development has been extensively studied, with PAX4 expression being enriched during the embryonic period and markedly reduced after birth, its function in mature β cells is not yet well understood [7]. Recent studies showed the presence and function of PAX4 in adult islets [8–11]. Pax4 is induced upon a high-fat diet (HFD) or metabolic stress via Srebp1c, protects β cells from stress-induced apoptosis by suppressing selective nuclear factor-kB target genes, and promotes β cell proliferation [8, 12–14]. However, the functional investigation on the PAX4 R192H variant remain limited. Structural analyses of PAX4 R192H suggest that this mutation impairs DNA binding, indicating a potential loss-of-function [15]. Recently, Lau et al. investigated the role of PAX4 R192H in human induced pluripotent stem cell (hiPSC)-derived β cells and found increased formation of polyhormonal endocrine cells along with reduced insulin content [16]. However, the in vivo consequences of the *PAX4* R192H variant and the effects of β cell-specific *Pax4* KO, remain unexplored.

In this study, we generated and characterized *PAX4* R192H knock-in (*Pax4*^R192H/R192H^) and inducible β cell-specific *Pax4* KO (*Pdx1-Cre*^*ERT2*^;*Pax4*^floxed/floxed^) mouse models to investigate the underlying mechanism of increased type 2 diabetes risk and β cell dysfunction in *PAX4* R192H. While *Pax4* R192H mice show normal metabolic phenotypes under standard chow diet (SCD), they exhibit impaired β cell compensation, reduced insulin secretion, and increased endoplasmic reticulum (ER) stress under HFD conditions. An inducible β cell-specific *Pax4* KO model confirmed a more severe phenotype, reinforcing the essential role of Pax4 in adult β cell function. Furthermore, we used a 14-year prospective human cohort that underwent biennial oral glucose tolerance tests and demonstrated that individuals carrying the *PAX4* R192H variant have an accelerated decline in β cell function (disposition index), particularly when combined with obesity or additional genetic predisposition to diabetes, highlighting the variant’s impact on β cell compensatory capacity under metabolic stress. Our findings underscore the importance of PAX4 in preserving β cell identity and function in the pathogenesis of type 2 diabetes and provide mechanistic insights into how the *PAX4* R192H variant compromises β cell compensation under metabolic stress.

## Methods

### Animal experiments

All mice were housed and bred in a specific-pathogen-free facility at Korea Advanced Institute of Science and Technology Laboratory Animal Resource Center with a 12-h light and 12-h dark cycle (light on at 7:00 am) at room temperature (22℃ ± 2℃) and 50% ± 10% humidity conditions. Only male mice were used in this study. A SCD (Purina 5008 chow) or HFD (Research Diet, #D12492; 60% fat) and water were given ad libitum. All animal experiments were approved by the Institutional Animal Care and Use Committee of Korea Advanced Institute of Science and Technology and conducted in accordance with relevant guidelines and regulations (KA2025-102-v1).

We generated a mutant mouse that harbors R200H mutation in the endogenous *Pax*4 allele corresponding to the R192H variant in humans. *Pax4*^WT/WT^ (wildtype) and *Pax4*^R192H/R192H^ (*Pax4* R192H) littermate mice obtained by crossing *Pax4*^WT/R192H^ were used in the study. All experiments involving *Pax4* R192H mice were performed using homozygous knock-in *Pax4* R192H mice of C57BL6/N background.

Sperm from mice carrying a *Pax4*^*tm1a*^ allele (*Pax4*^*tm1a(EUCOMM)Hmgu*^, MGI:4841624) was obtained from the European Mouse Mutant Archive (EMMA) and used for in vitro fertilization. *Pax4*^*tm1a*^ allele contains FRT sites, lacZ, loxP sites, and neomycin resistance cassettes inserted in the intron located between exons 1 and 2. Mice harboring a *Pax4*^*tm1a*^ allele were crossed with ACTFLPe mice [17] (MGI: 3714491) to remove both lacZ and neomycin resistance cassettes, generating mice harboring *Pax4*^*tm1c*^ allele. For brevity, mice carrying *Pax4*^*tm1a*^ or *Pax4*^*tm1c*^ allele are referred to as *Pax4*^*LacZ*^ and *Pax4*^*floxed*^ mice. To generate *Pax*4 β cell specific inducible KO mice (*Pax4* βiKO), *Pax4* homozygote floxed (*Pax4*^floxed/floxed^) mice (MGI:4841624) were crossed with *Pdx1-Cre*^*ERT2*^ mice (MGI: 26844321) [18]. Cre recombination was induced by five consecutive daily intraperitoneal injections of tamoxifen (75 g/kg; T5648, Sigma-Aldrich) dissolved in corn oil (Corn oil, C8267, Sigma-Aldrich). *Pax4*^flox/flox^; *Pdx1-Cre*^*ERT2*^ (-) mice treated with tamoxifen were used as the control group.

### Genotyping

Genomic DNA was extracted from tail of *Pax4* R192H mice for genotyping. For *Pax4* R192H, PCR was performed using forward primer and reverse primer (Forward: 5′-CCTCTGCTGCCATCTCTACC-3′; Reverse: 5′-CTTCCCAGTCCCCACAGTAA-3′), followed by overnight incubation at 37 ℃ with the restriction enzyme, Nco1 (Enzynomics, ATG-R004S). If the DNA was cut, the banding size was (152 and 372 bp); if not cut, the banding size was (527 bp), used for genotyping. Genomic DNA was extracted from tail of *Pax4* βiKO mice for genotyping. The *Pax4*^floxed/floxed^ was detected by PCR using the primers: forward, 5′-GGACTGTCCAACAGTGAAAC-3′; reverse, 5′-CACTAACATCACAGAGGCAGCTGAG-3′. For *Pax4* βiKO mice, the *Pdx1-Cre*^*ERT2*^ transgene was detected using the primers: forward, 5′-CTGGACTACATCTTGAGTTGC-3′; reverse, 5′-GGTGTACGGTCAGTAAATTTG-3′. PCR was performed using Taq polymerase (NanoHelix) with the following conditions: 95 °C for 5 min, followed by 35 cycles of 95 °C for 30 s, 58 °C for 30 s, and 72 °C for 45 s, with a final extension at 72 °C for 7 min. PCR products were analyzed using 2% agarose gel electrophoresis.

### Metabolic assays

Body weight and random blood glucose levels were measured weekly. For the glucose tolerance test (GTT), the mice were fasted overnight for 16 h and 2 g/kg D-glucose (Sigma) was administered via intraperitoneal injection. For glucose measurement, blood glucose levels from 0, 15, 30, 60, 90, and 120 min after injection were measured from tail vein using a glucometer (Gluco Dr.TOP glucometer, Allmedicus). For insulin tolerance test, mice were fasted for 6 h and injected with human insulin (Humulin R, Lilly) in phosphate-buffered saline (PBS; 0.75U/kg for SCD or 1 U/kg for HFD). Blood samples were then obtained from the tail vein at 0, 15, 30, 45, 60, 75, and 90 min after injection.

### Glucose-stimulated insulin secretion

For in vivo glucose-stimulated insulin secretion (GSIS), mice fasted for 16 h were given an intraperitoneal injection of D-glucose in PBS (2 g/kg for SCD and 3 g/kg for HFD). Blood samples were collected at 0, 15 and 30 min after the injection using heparinized capillary tubes (Marienfeld). Subsequently, the samples were centrifuged at 1500 g for 10 min at 4℃ to obtain the supernatant, which was then used for insulin ELISA analysis (80-INSMSU-E01, ALPCO) as previously described [19].

For ex vivo GSIS, isolated islets from mice as described previously [20] were incubated overnight in Roswell Park Memorial Institute (RPMI) 1640 medium containing 10% fetal bovine serum and 1% penicillin/streptomycin at 37℃ in a humidified incubator. The islets were transferred to Krebs–Ringer HEPES (KRH) buffer containing 5 mM glucose, incubated for 2 h at 37℃. The islets were then transferred to 2.8 mM glucose KRH buffer and incubated for 1 h. After incubation, the islets were hand-picked (n = 10 islets each) and placed into a 12-well non-coated dish. To measure the basal insulin secretion levels, the samples were incubated with 2.8 mM glucose KRH buffer for 15 min, and 50 μL of supernatant was obtained for insulin measurements. For high glucose stimulation, islets isolated from mice fed a SCD were incubated in KRH buffer containing 16.8 mM glucose for 15 min, and the supernatants were collected. Additionally, islets isolated from mice fed a HFD were incubated in KRH buffer containing 20 mM glucose for 15 min, and the supernatants were collected. For intracellular insulin extraction, the islets were incubated in acid–ethanol (1.5% HCL in 100 mL of 70% ethanol) for 16 h at 4℃. The same volume of 1 M Tris–Cl buffer (pH 8.0) was added for neutralization. Insulin levels were measured using a mouse insulin ELISA kit (80-INSMSU-E01, ALPCO). Insulin secretion was calculated by dividing the secreted insulin by the insulin content extracted from the islets.

### Immunofluorescence staining and quantification of cells

Pancreata from mice were perfused with PBS, fixed in 10% neutral buffered formalin (HT501128, Sigma-Aldrich) for 4 h at room temperature and washed with deionized water for 1 h. The tissues were processed using an automatic tissue processor (TP1020, Leica Biosystems) and embedded in molten paraffin wax. Paraffin-embedded tissue sections were sliced at 4-μm thickness and mounted on adhesive glass slides (081000, Marienfeld). The slides were deparaffinized and rehydrated. Antigen retrieval was performed by incubating the slides in sodium citrate buffer (10 mM sodium citrate, pH 6.0) for 13 min at 95℃. The slides were washed in PBS for 10 min and samples were blocked with 4% normal donkey serum (017–000–121, Jackson ImmunoResearch) in PBS for 30 min at room temperature. The samples were then incubated for 16 h at 4℃ with primary antibodies. The samples were washed in PBS for 5 min and incubated for 1 h at room temperature with secondary antibodies. The samples were washed in PBS for 5 min, incubated for 5 min with DAPI (D9542, Sigma-Aldrich, 1 μg/mL) at room temperature, and then mounted with fluorescence mounting medium (S3023, Dako). Images were acquired using a LSM 780 confocal microscope (Carl Zeiss).

The following antibodies were used: guinea pig anti-Insulin (1:500, Dako, A0564); mouse anti-Glucagon (1:500, Sigma- Aldrich, G2654); rabbit anti-Somatostatin (1:500, Dako, A0566); mouse anti-Pdx1 (1:500, DSHB, F6A11); rabbit anti-MafA (1:1000, CST, 79,737); rabbit anti-Glut2 (1:500, Merck Millipore, 07–1402-I); rabbit anti-Ki67 (1:1000, Abcam, ab15580); rabbit anti-Ngn3 (1:50, Abcam, AB38548); and rabbit anti-Ucn3 (1:500, Pheonix, H-019–29).

The following secondary antibodies were used: Alexa Fluor 488 conjugated anti-guinea pig IgG (1:1000, Jackson ImmunoResearch, 706–545-148); Alexa Fluor 594 conjugated anti-rabbit IgG (1:1000, Jackson ImmunoResearch, 711–585-152); and Alexa Fluor 594 conjugated anti-mouse IgG (1:1000, Jackson ImmunoResearch, 715–585-151).

Images were acquired using a confocal microscope (LSM 780 confocal, Carl Zeiss) under consistent acquisition settings. Quantification of α cells or δ cells was performed using ImageJ 1.53e by manually counting the number of glucagon-, somatostatin-, and insulin-positive cells in each islet. The proportion of α cells was calculated as the number of glucagon-positive cells divided by (glucagon-positive + insulin-positive cells), and the proportion of δ cells was calculated as the number of somatostatin-positive cells divided by (somatostatin-positive + insulin-positive cells), both expressed as a percentage. A minimum of 1000 endocrine cells per mouse from at least three biological replicates were analyzed. Similarly, the quantification of GLUT2 and MAFA positive area was conducted using an analogous approach, analyzing more than 100 islets per mouse to ensure statistical robustness.

Pancreas sections on slides (eight per mouse, taken at least 100 μm apart) were subjected to insulin and Ki67 immunofluorescence staining. The β cell proliferation rate was calculated as the percentage of insulin and Ki67-copositive cells over all insulin positive cells.

### Immunohistochemistry staining and β cell area

Formalin-fixed, paraffin-embedded pancreatic sections were deparaffinized and rehydrated. For Immunohistochemistry staining, antigen retrieval was performed as described for immunofluorescence staining. The slides were cooled and washed in PBS for 10 min and endogenous peroxidases were blocked with BLOXALL solution (SP-6000, Vector Laboratories) for 10 min at room temperature. The slides were blocked with 2% normal goat serum (S-1000, Vector Laboratories) in PBS for 1 h at room temperature, and then incubated for 16 h at 4℃ with primary INS antibody (A0564, Dako, 1:500). The slides were washed in PBS for 10 min and stained using an ABC-HRP kits (PK-4001/4007, Vector Laboratories) as directed. Subsequently, 3,3′-Diaminobenzidine (SK-4100, Vector Laboratories) was used as a substrate for HRP enzyme. The slides were mounted. Images were acquired using a slide scanner. Pancreas sections on slides (6 per mouse, taken at least 100 μm apart) were subjected to insulin IHC staining. Whole-pancreas images were acquired using a JuLI Stage recorder (NanoEnTek). The insulin-positive area and the pancreas images were measured using ImageJ software (NIH). The β cell area was calculated by dividing the insulin-positive area by the pancreatic area.

### X-gal staining on Pax4^LacZ^ islets

Islets isolated from 12-week-old male *Pax4*^*LacZ/*+^ mice were incubated overnight in RPMI 1640 medium containing 10% fetal bovine serum and 1% penicillin/streptomycin at 37℃ in a humidified incubator. Islets were then exposed to 4 nM recombinant human Activin A (338-AC, R&D Systems) for 72 h. For X-gal staining, islets were fixed in fixative solution (2% formaldehyde (P6148, Sigma) and 0.2% glutaraldehyde (16,210, Electron Microscopy Sciences) in PBS) for 15 min on ice. After washing in PBS, islets were incubated in X-gal staining solution (1 mg/ml X-Gal (XF2001, Biosesang), 5 mM potassium ferricyanide, 5 mM potassium ferrocyanide, 2 mM MgCI_2_, 0.01% sodium deoxycholate, and 0.02% Nonidet P-40 (NP-40) in PBS) overnight at room temperature. Following washing in PBS, images were analyzed using a stereomicroscope (SZ61, Olympus).

### Quantitative RT–PCR

Total RNA was extracted from isolated pancreatic islets using TRIzol (Invitrogen) according to the manufacturer’s instructions. One microgram of total RNA was reverse-transcribed using a High-Capacity cDNA Reverse Transcription Kit (Applied Biosystems). Quantitative RT–PCR was performed using Fast SYBR Green Master Mix (Applied Biosystems) on a ViiA 7 Real-Time PCR System (Applied Biosystems). Primer sequences were as follows: *Pax4* forward-1, *5*′-AAGGCTCCCAGTGTGTCCTCTA*−3*′; *Pax4* reverse-1*, 5*′-GATAGTCCGATTCCTGTGGCTG−*3*′; *Pax4* forward-2*, 5*′-AAGGCTCCCAGTGTGTCCTCTA−3′; *Pax4* reverse-2, *5*′-GTGATCTGAGTTGAGTCCAGTGCA−3′; *Actb* forward, *5*′-CAGCTTCTTTGCAGCTCCTT−*3*′; *Actb* reverse, *5*′-CTTCTCCATGTCGTCCCAGT−3′. Relative gene expression levels were calculated using the ddCt method, with *Actb* serving as an internal control.

### RNA-sequencing analysis

Total RNAs from isolated pancreatic islets were extracted using TRIzol (15596–026, Invitrogen) following the manufacturer’s protocol. RNA-seq libraries were prepared using TruSeq Stranded Total RNA LT Sample Prep Kit (Illumina) and sequenced (paired-end 100-bp reads) using an Illumina NovaSeq 6000 platform. The reads were aligned to mouse genome (mm10) using STAR (v2.7.1) [21]. Transcript abundance calculated by RSEM (v1.3.3) [22] was summarized to gene-level by the tximport R package (v1.36.0) [23]. Differential gene expression analysis was performed using the DESeq2 R package (v1.48.1) [24] with default parameters. DEGs were clustered by K-means clustering with the optimal number of clusters determined by the fviz_nbclust function in Factoextra R package (v1.0.7). Functional enrichment analyses of DEGs were performed using clusterProfiler R package (v4.16.0) [25].

### Droplet-based single-cell RNA sequencing (scRNA-seq)

Pancreatic islets isolated from two to three mice were pooled and digested in 0.25% trypsin with EDTA (25200–056, Gibco) at 37℃ for 10 min with gentle pipetting every 3 min. Dissociated cells were washed and resuspended with PBS containing 0.04% BSA (160069, MP Biomedicals) and loaded onto the 10 × Genomics Chromium system targeting 5,000 cells. Libraries for scRNA-seq were generated using the Chromium Next GEM Single Cell 3′ Reagent Kits v3.1. Libraries were sequenced using an Illumina HiSeqX platform.

### Preprocessing of scRNA-seq data

scRNA-seq data were processed using the Cell Ranger software (v6.0; 10 × Genomics). Alignment of reads to mouse reference genome (GRCm38) and generation of gene expression matrices were performed by cellranger count pipeline. Filtered expression matrices were loaded by the CreateSeuratObject function of the Seurat R package (v5.2.0) [26]. Ambient RNAs in droplet were estimated and removed by the SoupX R package (v1.6.2) [27]. Cells that meet the following criteria were retained: (1) low fraction of mitochondrial genes (< 10%); (2) UMI counts between 3000 and 50,000; or (3) features between 2000 and 5000. Furthermore, putative doublets were detected and removed using the DoubletFinder R package (v2.0.6) [28]. Preprocessed datasets from SCD-fed wildtype, SCD-fed *Pax4* R192H, HFD-fed wildtype, and HFD-fed *Pax4* R192H islets were merged by the merge R function.

### Analysis of scRNA-seq data

Data analysis was performed using the Seurat R package (v5.2.0) [26]. Normalization and variance stabilization was performed using the SCTransform function, with regression of mitochondrial gene content. After running PCA by the RunPCA function, integration was performed by the IntegrateLayers function using Harmony methods [29]. For a graph-based cell clustering, the FindNeighbors and FindClusters functions were used with the first 30 principal components. Followed by dimensional reduction using the RunUMAP function with the first 30 principal components, cells were visualized on the two-dimensional UMAP plot. DEGs for each cluster were identified with the FindMarkers function using the MAST R package [30]. Cell clusters were annotated based on the expression of canonical endocrine cell marker genes. Subset of clusters identified as endocrine cells were re-processed using the same workflows as described above. Functional enrichment analyses of DEGs were performed using the clusterProfiler R package (v4.16.0) [25]. Module scores for gene sets were obtained by using the AddModuleScore function of the Seurat R package, which calculate average expression levels of gene sets imported from the Molecular Signatures Database (MSigDB; v24.1.0) [31–33]. β cell signature gene set was generated by combining genes of ‘Hallmark pancreas beta cells (MM3909)’ of MSigDB and putative markers of mature β cell.

### Human cohort

#### Study population

The Ansan-Ansung Cohort Study is a prospective, community-based cohort study in South Korea that has been previously described in detail [34, 35]. The study is part of the Korean Genome and Epidemiology Study which aimed to investigate trends and the genetic and environmental etiology of chronic complex disorders including diabetes. Participants aged 40–69 years, residing either in the urban Ansan or the rural Ansung community, were enrolled in the study. The baseline survey was done between 2001 and 2002, and the follow-up examination was performed every 2 years. Data from 2001 to 2016 were included for analysis in this study. The study protocol was approved by the ethics committee of the Korean Center for Disease Control and the institutional review board of Seoul National University Hospital (1801–095–916). All participants provided written informed consent.

### Procedures

Details of the procedures were described previously [34, 35]. Briefly, anthropometric parameters were measured using standard methods. Fasting plasma glucose (FPG), fasting insulin and HbA1c were measured in a central laboratory following an overnight 12-h fast. Each participant underwent a 2-h 75 g OGTT at study enrollment and the test was repeated every 2 years. Plasma samples were taken at 0, 60, and 120 min during the OGTT to measure plasma glucose and insulin concentrations. Participants who had been previously diagnosed with diabetes or reported taking diabetic medication were exempted from undergoing OGTT. Definition of diabetes were based on the American Diabetes Association diagnostic criteria using FPG, 2-h glucose, and HbA1c [36].

Pancreatic β cell secretory response was estimated by 60-min insulinogenic index (IGI_60_), which was calculated based on plasma insulin and glucose levels at 0 and 60 min during the OGTT. IGI_60_ was calculated using the following formula:

$$\frac{{\mathrm{i}\mathrm{n}\mathrm{s}\mathrm{u}\mathrm{l}\mathrm{i}\mathrm{n}}_{60\mathrm{m}\mathrm{i}\mathrm{n}}-{\mathrm{i}\mathrm{n}\mathrm{s}\mathrm{u}\mathrm{l}\mathrm{i}\mathrm{n}}_{0\mathrm{m}\mathrm{i}\mathrm{n}} [\upmu \mathrm{U}/\mathrm{m}\mathrm{L}]}{{\mathrm{g}\mathrm{l}\mathrm{u}\mathrm{c}\mathrm{o}\mathrm{s}\mathrm{e}}_{60\mathrm{m}\mathrm{i}\mathrm{n}}-{\mathrm{g}\mathrm{l}\mathrm{u}\mathrm{c}\mathrm{o}\mathrm{s}\mathrm{e}}_{0\mathrm{m}\mathrm{i}\mathrm{n}} [\mathrm{m}\mathrm{m}\mathrm{o}\mathrm{l}/\mathrm{L}]}$$

Insulin sensitivity index (ISI) was assessed using the Matsuda index, calculated from values at 0, 60, and 120 min of the OGTT as follows:

$$\frac{10000}{\sqrt{\left(Fasting\:Glucose\right)\times (Fasting\:Insulin)\times (Mean\:Glucose)\times (Mean\:Insulin)}}$$

To reflect β cell function while accounting for insulin sensitivity, we estimated the OGTT-derived DI by multiplying IGI_60_ with ISI.

Participants were genotyped using the KoreanChip [37, 38], a microarray chip optimized for the Korean population that includes a custom-designed probe targeting the *PAX4* R192H variant (rs2233580), enabling its direct genotyping. Imputation was done using the 1000G reference panel [39]. As we previously reported, there is a low-frequency nonsynonymous variant (chr7:127253551, rs3824004, effect allele T), independent from *PAX4* R192H, that results in a different amino acid change in the *PAX4* 192 codon (Arg192Ser) [5]. We removed individuals with this variant to directly compare Arg and His in the *PAX4* 192 codon (*N* = 444).

Among the 5,493 participants with genotyped with KoreanChip in the Ansan-Ansung Cohort Study, 5,049 participants did not have the rs3824004 variant, 4,369 participants were not diagnosed of type 2 diabetes at baseline, and 4,242 participants with at least 2 points of OGTT data during follow-up were included in the present analysis.

### Trajectory analysis

ISI, IGI_60_, and DI were normalized using logarithmic transformation due to their non-Gaussian distribution. Participants were grouped depending on the *PAX4* R192H variant. We conducted a longitudinal analysis of log_2_-transformed IGI_60_, ISI, and DI using linear mixed-models to estimate the average levels of the parameters over time within genetic risk groups from baseline to up to 14 years of follow-up [40]. The fixed effects were time, group, and group-by-time, and individuals were included as a random effect. The primary analysis included adjustment for age at enrollment, sex, BMI, and the first 10 principal components of ancestry. OGTT data were used until the diagnosis of diabetes as treatment for diabetes could significantly alter OGTT results.

### Construction of genetic risk score for type 2 diabetes

We calculated the genetic predisposition to type 2 diabetes by calculating genetic risk score (GRS) restricted to significant variants and effect sizes from the T2DGGI consortium^2^. Index variants with low minor allele frequency (< 1%) and poor imputation quality (*r*^2^ < 0.7) were excluded from the analysis (criteria 1). Additionally, to construct a GRS independent from the *PAX4* R192H variant, we excluded index variants that are within 500 kb and are in linkage disequilibrium (*r*^2^ < 0.05) with the variant (criteria 2). From the 1,289 genome-wide significant index variants, a total of 1,035 variants passed the first criteria in our imputed genetic dataset and five variants were excluded due to the second criteria. Total of 1,030 variants and their effect sizes for East Asians were used to construct GRS for type 2 diabetes.

### Statistical analysis

All data are presented as the mean ± standard error of measurements for continuous variables or as a number (percentage) for nominal variables. The statistical significance was obtained using Student’s *t*-test (two-tailed) unless stated otherwise. Parts of the statistical analysis were performed with Python version 2.7.5 and R software version 4.2.2 using the computing server at the Genomic Medicine Institute Research Service Center. A *P* value < 0.05 was considered statistically significant.

## Results

### Pax4 variant does not alter metabolic phenotypes in mice fed a standard chow diet

**Fig. 1:**
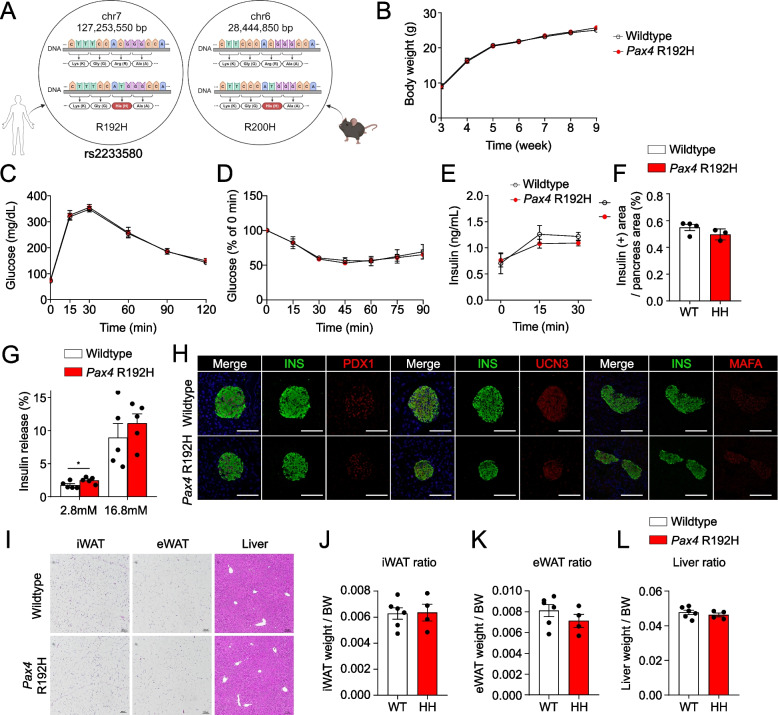
*Pax4* R192H variant does not alter metabolic phenotypes in mice fed a standard chow diet. **A** Knock-in mouse model of *Pax4* R192H. **B** Body weight of mice fed a standard chow diet; *n* ≥ 5 per group. **C** Intraperitoneal glucose tolerance test in wildtype and *Pax4* R192H (12-week-old) mice after 16-h fasting; n ≥ 9 per group. **D** Intraperitoneal insulin tolerance test in wildtype and *Pax4* R192H (12-week-old) mice after 6-h fasting; *n* ≥ 9 per group. **E** In vivo glucose-stimulated insulin secretion in wildtype and *Pax4* R192H (12-week-old) mice after 16-h fasting; *n* ≥ 4 per group. **F** Quantification of insulin positive area as a percentage of total pancreatic area in 10-week-old wildtype and *Pax4* R192H mice fed a standard chow diet; *n* ≥ 3 per group. **G** Ex vivo glucose-stimulated insulin secretion in islets isolated from wildtype and *Pax4* R192H (12-week-old) mice. Islets were pooled from 2–3 mice per preparation; *n* = 5 independent pooled samples per group. **H** Immunofluorescence staining of β cell markers (PDX1, UCN3 and MAFA) and insulin in pancreas of mice (12-week-old) fed a standard chow diet; *n* = 4 per group. **I** Representative histological images of inguinal white adipose tissue (iWAT), epididymal white adipose tissue (eWAT), and liver from 12-week-old wildtype and *Pax4* R192H mice stained with hematoxylin and eosin (H&E); *n* = 4 per group. **J**–**L** Adipose tissue-to-body weight and liver-to-body weight ratios of iWAT (**J**), eWAT (**K**), and liver (**L**); *n* = 4–6 per group. *Pax4* R192H refers to the homozygous knock-in mice. BW, body weight; WT, wildtype; HH, *Pax4* R192H homozygous knock-in

To investigate the mechanism how *PAX4* R192H variant increases the risk of diabetes, we generated a mutant mouse carrying an R200H substitution in the endogenous *Pax4* allele, corresponding to the human R192H variant. Mice homozygous for this variant are hereafter referred to as the *Pax4* R192H mouse (Fig. 1A). We did not detect major disturbances in endocrine cell development or in the expression of key endocrine markers (PDX1, NGN3, INS, GCG and SST) in *Pax4* R192H mice during embryonic development (Supplementary Fig. 1A–C).

Although Pax4 is highly expressed in embryonic pancreas and its expression is markedly reduced after birth, multiple lines of evidence suggest functional role of Pax4 in adult islets [8, 12–14]. Indeed, quantitative RT–PCR analysis showed low but measurable *Pax4* mRNA expression in mouse islets (Supplementary Fig. 2 A). Furthermore, X-gal staining in *Pax4*^*LacZ/*+^ reporter mice revealed Pax4-expressing cells in adult islets with a heterogeneous expression pattern which was further stimulated by Activin A, a known mitogenic factor reported to upregulate Pax4 expression (Supplementary Fig. 2B). *Pax4* mRNA levels were comparable between wildtype control and *Pax4* R192H mice (Supplementary Fig. 2 C).

To explore whether the R192H variant leads to metabolic alterations over time, we longitudinally assessed the metabolic phenotypes of the mice. During growth, no significant differences in body weight were observed between *Pax4* R192H and wildtype mice on an SCD (Fig. 1B). *Pax4* R192H and wildtype mice at 4 and 6 weeks of age showed similar glucose tolerance (Supplementary Fig. 2D, E). Glucose tolerance and insulin sensitivity were comparable between 12-week-old *Pax4* R192H and wildtype mice (Fig. 1C, D). The β cell phenotypes including β cell area, in vivo glucose-stimulated insulin secretion, and β cell identity markers (PDX1, UCN3, MAFA) were largely comparable, although *Pax4* R192H islets exhibited a slight increase in basal insulin secretion (Fig. 1E–H). Other metabolic organs, including the liver and white adipose tissues, also showed comparable weight and histology between *Pax4* R192H and wildtype mice (Fig. 1I–L). These findings indicate that metabolic phenotypes, including glucose tolerance and β cell function, in *Pax4* R192H mice fed an SCD were comparable to those in wildtype mice.

### Pax4 variant impairs glucose tolerance and β cell function in mice under metabolic stress

**Fig. 2:**
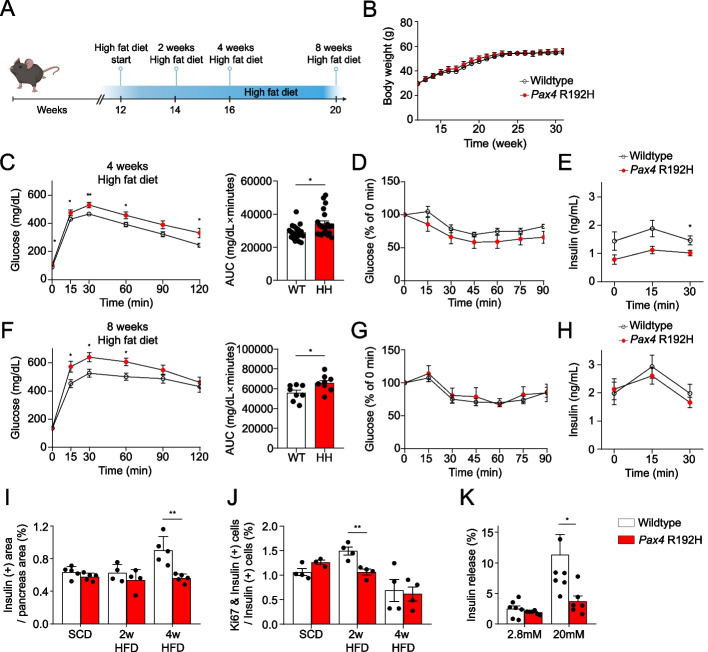
*Pax4* R192H variant impairs glucose tolerance and β cell function in mice under metabolic stress.** A** Scheme of the experimental protocol used in this study. **B** Body weight of mice fed a high-fat diet; *n* ≥ 5per group. **C** Intraperitoneal glucose tolerance test and area under the curve (AUC) in wildtype and *Pax4* R192H mice fed a high-fat diet for 4 weeks (16-week-old) after 16-h fasting; n ≥ 20 per group. **D** Intraperitoneal insulin tolerance test in wildtype and *Pax4* R192H mice fed a high-fat diet for 4 weeks (16-week-old) after 6-h fasting; *n* ≥ 6 per group. **E** In vivo glucose-stimulated insulin secretion in wildtype and *Pax4* R192H mice fed a high-fat diet for 4 weeks (16-week-old) after 16-h fasting; *n* ≥ 6 per group. **F** Intraperitoneal glucose tolerance test and AUC in wildtype and *Pax4* R192H mice fed a high-fat diet for 8 weeks (20-week-old) after 16-h fasting; n ≥ 5 per group. **G** Intraperitoneal insulin tolerance test in wildtype and *Pax4* R192H mice fed a high-fat diet for 8 weeks (20-week-old) after 6-h fasting; *n* ≥ 6 per group. **H** In vivo glucose-stimulated insulin secretion in wildtype and *Pax4* R192H mice fed a high-fat diet for 8 weeks (20-week-old) after 16-h fasting; *n* = 7 per group. **I** Quantification of insulin positive area as a percentage of total pancreatic area in wildtype and *Pax4* R192H mice fed a standard chow diet (12-week-old), 2 weeks of a high-fat diet (14-week-old), or 4 weeks of a high-fat diet (16-week-old); *n* ≥ 4 per group. **J** Quantification of Ki67 and insulin positive cells as a percentage of total insulin positive cells in the pancreas of mice fed a standard chow diet (12-week-old), 2 weeks of a high-fat diet (14-week-old), or 4 weeks of a high-fat diet (16-week-old); *n* = 3–4 mice per group. **K** Ex vivo glucose-stimulated insulin secretion in islets from wildtype and *Pax4* R192H mice fed a high-fat diet for 4 weeks (16-week-old). Islets were pooled from 2–3 mice per preparation; *n* = 6 independent pooled samples per group. *Pax4* R192H refers to the homozygous knock-in mice. WT, wildtype; HH, *Pax4* R192H homozygous knock-in; SCD, standard chow diet; HFD, high-fat diet. *P < 0.05; **P < 0.01

To investigate whether the PAX4 variant affects metabolic adaptations under conditions of metabolic stress, *Pax4* R192H and wildtype mice were fed an HFD from 12 weeks of age (Fig. 2A). No significant differences in body weight were observed between the two groups (Fig. 2B). After 4 weeks of an HFD, *Pax4* R192H mice exhibited impaired glucose tolerance and reduced insulin secretory function without changes in insulin sensitivity compared with those in wildtype controls (Fig. 2C–E). Prolonged exposure to an HFD up to 8 weeks resulted in further deterioration of glucose tolerance in *Pax4* R192H mice, while insulin sensitivity and insulin secretory function were comparable between the groups, and plasma insulin levels were already elevated to near-maximal levels (Fig. 2F–H). In addition, no significant differences were observed in liver and white adipose tissues between the groups after HFD (Supplementary Fig. 3A–D). These data suggest that β cell compensation in response to metabolic stress is transiently impaired in *Pax4* R192H mice despite experiencing a similar degree of metabolic challenges as wildtype mice.

We further investigated β cell phenotypes of *Pax4* R192H mice under an HFD. *Pax4* mRNA expression levels in pancreatic islets were comparable under an HFD (Supplementary Fig. 3E). While pancreatic β cell area was comparable between genotypes under an SCD, *Pax4* R192H mice failed to exhibit the early expansion of β cell area during the initial 4 weeks of an HFD, resulting in a reduced β cell area compared to wildtype mice (Fig. 2I and Supplementary Fig. 3 F). Consistently, β cell proliferation significantly increased in wildtype mice after 2 weeks of HFD, whereas *Pax4* R192H mice did not exhibit induction of β cell proliferation (Fig. 2J and Supplementary Fig. 3G). Furthermore, glucose-stimulated insulin secretion was significantly impaired in islets isolated from *Pax4* R192H mice after 4 weeks of an HFD (Fig. 2K). Despite these functional deficits, immunofluorescence staining showed no notable differences in the expression of key β cell markers, including PDX1, GLUT2, MAFA and insulin, between the groups (Supplementary Fig. 3H). Overall, these findings indicate that *Pax4* R192H mice have impaired β cell compensatory capacity in response to metabolic stress with reduced β cell area expansion and insulin secretory function after an HFD.

### Pax4 variant disrupts β cell transcriptional adaptation to metabolic stress by promoting loss of β cell identity and stress response pathways

**Fig. 3:**
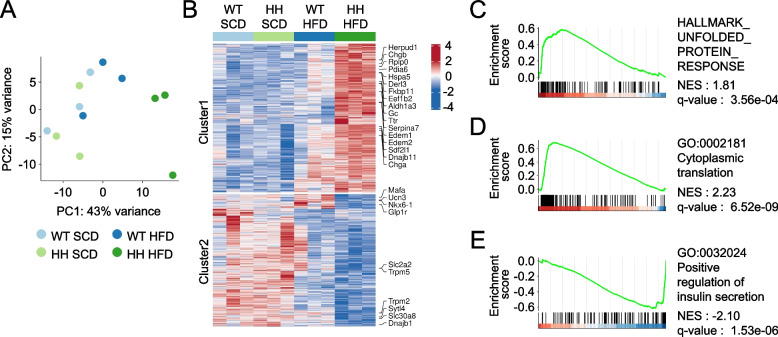
*Pax4* R192H variant disrupts β cell transcriptional adaptation to metabolic stress by promoting loss of β cell identity.** A** Principal component analysis (PCA) of bulk RNA-seq data from isolated islets of wildtype and *Pax4* R192H mice fed a standard chow diet (12-week-old) or high-fat diet (16-week-old). **B** Heatmap of k-means clustering of differentially expressed genes across all groups revealed two major clusters. **C**–**E** GSEA enrichment plots showing enrichment of genes in HFD-fed *Pax4* R192H islets (versus wildtype) for gene sets termed ‘Hallmark unfolded protein response (MM3883)’ (**C**), ‘Cytoplasmic translation (GO:0002181)’ (**D**), and ‘Positive regulation of insulin secretion (GO:0032024)’ **E**. *n* = 3 per group. *Pax4* R192H refers to the homozygous knock-in mice. WT SCD, wildtype with stand chow diet; HH SCD, *Pax4* R192H homozygous knock-in with standard chow diet; WT HFD, wildtype with high-fat diet; HH HFD, *Pax4* R192H homozygous knock-in with high-fat diet

To elucidate the molecular mechanisms underlying impaired β cell adaptation in *Pax4* R192H mice under metabolic stress, we performed bulk RNA sequencing (RNA-seq) on pancreatic islets isolated from wildtype and *Pax4* R192H mice fed an SCD (12-week-old) and 4 weeks of an HFD (16-week-old). Principal component analysis (PCA) and *k*-means clustering revealed minimal transcriptional differences between wildtype and *Pax4* R192H mice under an SCD consistent with their comparable metabolic phenotypes (Fig. 3A, B). In contrast, islets from *Pax4* R192H mice fed an HFD for 4 weeks showed distinct transcriptomic pattern from those of HFD-fed wildtype mice, indicating substantial gene expression changes in response to metabolic stress (Fig. 3A, B). While only a few differentially expressed genes (DEGs) were detected under an SCD, an HFD led to a substantial increase in DEGs, highlighting the exacerbation of transcriptional alterations by metabolic stress (Supplementary Fig. 4 A, B). Gene set enrichment analysis revealed upregulation of gene sets related to unfolded protein response and cytoplasmic translation in HFD-fed *Pax4* R192H mice, suggesting increased ER stress (Fig. 3C, D and Supplementary Fig. 4 C). In contrast, gene sets related to insulin and peptide secretion, including markers of β cell maturity and function such as *Mafa*, *Ucn3*, *Nkx6-1*, *Slc2a2*, and *Trpm5*, were significantly downregulated in HFD-fed *Pax4* R192H mice (Fig. 3E and Supplementary Fig. 4D). Moreover, upregulation of β cell dedifferentiation markers (*Aldh1a3* and *Serpina7*), together with increased expression of α cell enriched genes (*Gc* and *Ttr*) [41–44], suggested alterations in mature β cell identity in HFD-fed *Pax4* R192H mice (Fig. 3B). Collectively, these findings indicate that the *Pax4* R192H variant compromises β cell adaptation to metabolic stress by enhancing ER stress, suppressing β cell function, and promoting the loss of β cell maturity.

### Single-cell RNA-seq reveals that the PAX4 variant induces loss of β cell identity and triggers ER stress

**Fig. 4:**
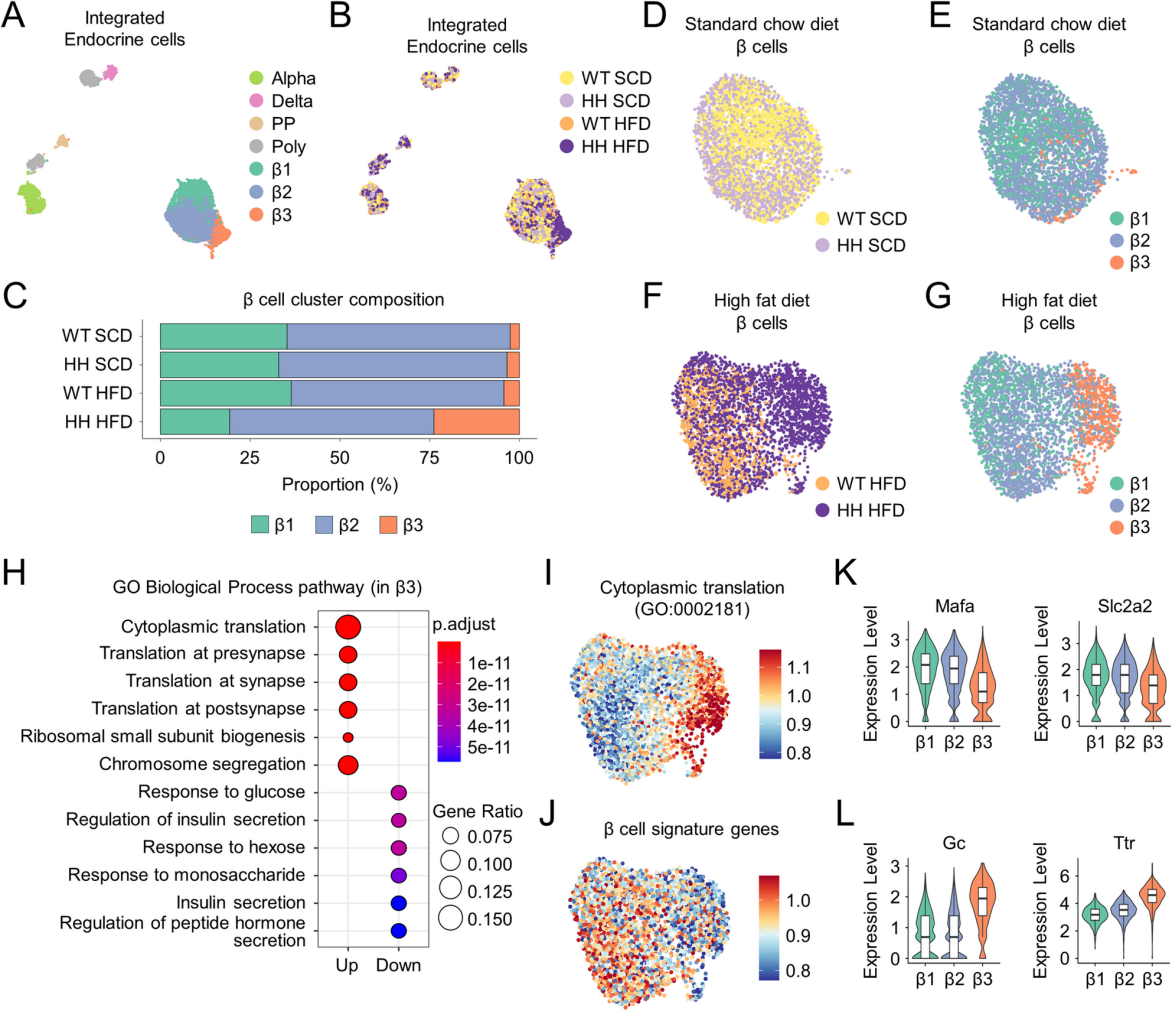
Single-cell RNA-seq reveals that the *Pax4* R192H variant induces loss of β cell identity with activation of translation process. **A**, **B** UMAP plot of pancreatic endocrine cells from wildtype and *Pax4* R192H mice fed standard chow diet (12-week-old) or high-fat diet (16-week-old). Colors represent cell types (**A**) or groups **B**. **C** Composition pattern of β cell clusters in wildtype and *Pax4* R192H mice fed standard chow diet (12-week-old) or high-fat diet (16-week-old). **D**, **E** UMAP plot of β cells from wildtype and *Pax4* R192H mice fed standard chow diet (12-week-old). Colors indicate groups (**D**) or cell types **E**. **F**, **G** UMAP plot of β cells from wildtype and *Pax4* R192H mice fed high-fat diet (16-week-old). Colors indicate groups (**F**) or cell types **G**. **H** Gene Ontology (GO) enrichment analysis of differentially expressed genes in β3 cluster compared to β1 and β2 clusters. **I**, **J** UMAP plot of β cells from wildtype and *Pax4* R192H mice fed high-fat diet (16-week-old) displaying module scores calculated using gene sets termed ‘Cytoplasmic translation (GO: 0002181)’ (**I**) and β cell signature gene set **J**. **K**, **L** Violin plot showing expression levels of *MafA, Slc2a2* (**K**), *Gc* and *Ttr* (**L**) among β cell clusters from wildtype and *Pax4* R192H mice fed high-fat diet (16-week-old). Islets were pooled from 2–3 mice. WT SCD, wildtype with standard chow diet; HH SCD, *Pax4* R192H homozygous knock-in with standard chow diet; WT HFD, wildtype with high-fat diet; HH HFD, *Pax4* R192H homozygous knock-in with high-fat diet

To understand the cellular heterogeneity and molecular alterations in islets at single-cell resolution, we performed single-cell RNA-seq (scRNA-seq) on islets isolated from wildtype and *Pax4* R192H mice fed either an SCD or an HFD. The transcriptional profiles were obtained from 9,903 endocrine cells that met the quality control criteria, including 5,320 cells from SCD-fed mice and 4,583 from HFD-fed mice. We identified nine distinct endocrine cell clusters by unsupervised clustering and annotated them based on canonical marker gene expression patterns: β (β1, β2, and β3; cluster 1, 2, and 3, respectively), α (clusters 4, 5), δ (cluster 7), pancreatic polypeptide-producing (cluster 9), and polyhormonal cells (cluster 6 and 8) (Supplementary Fig. 5A–D). The proportions and transcriptomic profiles of transcriptionally distinct subpopulations within α, δ, and PP cell lineages remained largely unchanged across genotypes or diet conditions (Fig. 4A, B and Supplementary Fig. 5E–H).

While β cell proportion in islets remained stable in wildtype or *Pax4* R192H mice fed an SCD, a distinct increase of β3 cluster was observed in HFD-fed *Pax4* R192H mice (Fig. 4C–G, and Supplementary Fig. 6 A). Differential gene expression analysis and gene set enrichment analysis revealed distinct transcriptomic signatures among β cell clusters (Fig. 4H and Supplementary Fig. 6B). Notably, β3 cluster showed significant upregulation of pathways related to cytoplasmic translation, ribosomal small subunit biogenesis, and chromosome segregation suggesting the enhanced translational or proliferative activity. Conversely, β3 cluster showed downregulation of genes involved in glucose responsiveness, insulin secretion, and peptide hormone secretion as well as reduced expression of β cell maturity markers (Fig. 4H–K and Supplementary Fig. 6B–D). Furthermore, β3 cluster demonstrated upregulation of β cell dedifferentiation markers (*Aldh1a3* and *Serpina7*) and ectopic expression of α cell enriched gene signatures (*Gc* and *Ttr*) (Fig. 4L and Supplementary Fig. 6D). This transcriptional features of β3 cluster closely resembled the pathway changes observed in the bulk RNA-seq analysis, further supporting the loss of mature β cell identity under metabolic stress in *Pax4* R192H mutants (Fig. 3 and Supplementary Fig. 4).

Next, we explored the role of *Pax4* in transcriptional regulation of β cell identity and function. Previously, Russ-Silsby et al. performed CUT&RUN assay by introducing V5-tagged *PAX4* in EndoC-βH1 human β cell line and identified 1,659 PAX4 binding peaks, suggesting PAX4 occupancies at regulatory elements related to pancreatic islet development and glucose-stimulated insulin secretion [45]. Comparison of PAX4 binding peaks with DEGs identified in β3 cluster of scRNA-seq data revealed an overlap of 61 genes (Supplementary Fig. 7 A), including *Glp1r*, *Cacna1d*, and *Itpr1*, which were downregulated in β3 cluster and were associated with β cell function (Supplementary Fig. 7B–D). These results suggest that *Pax4* is involved in transcriptional regulation of stress response and β cell function-associated genes. Collectively, these findings indicate that *Pax4* R192H variant compromises β cell identity and function under metabolic stress by promoting the loss of β cell maturity and enhancing ER stress-related transcriptional programs.

### Inducible knockout of Pax4 in adulthood results in more severe metabolic phenotypes compared with mice carrying the Pax4 missense mutation

**Fig. 5:**
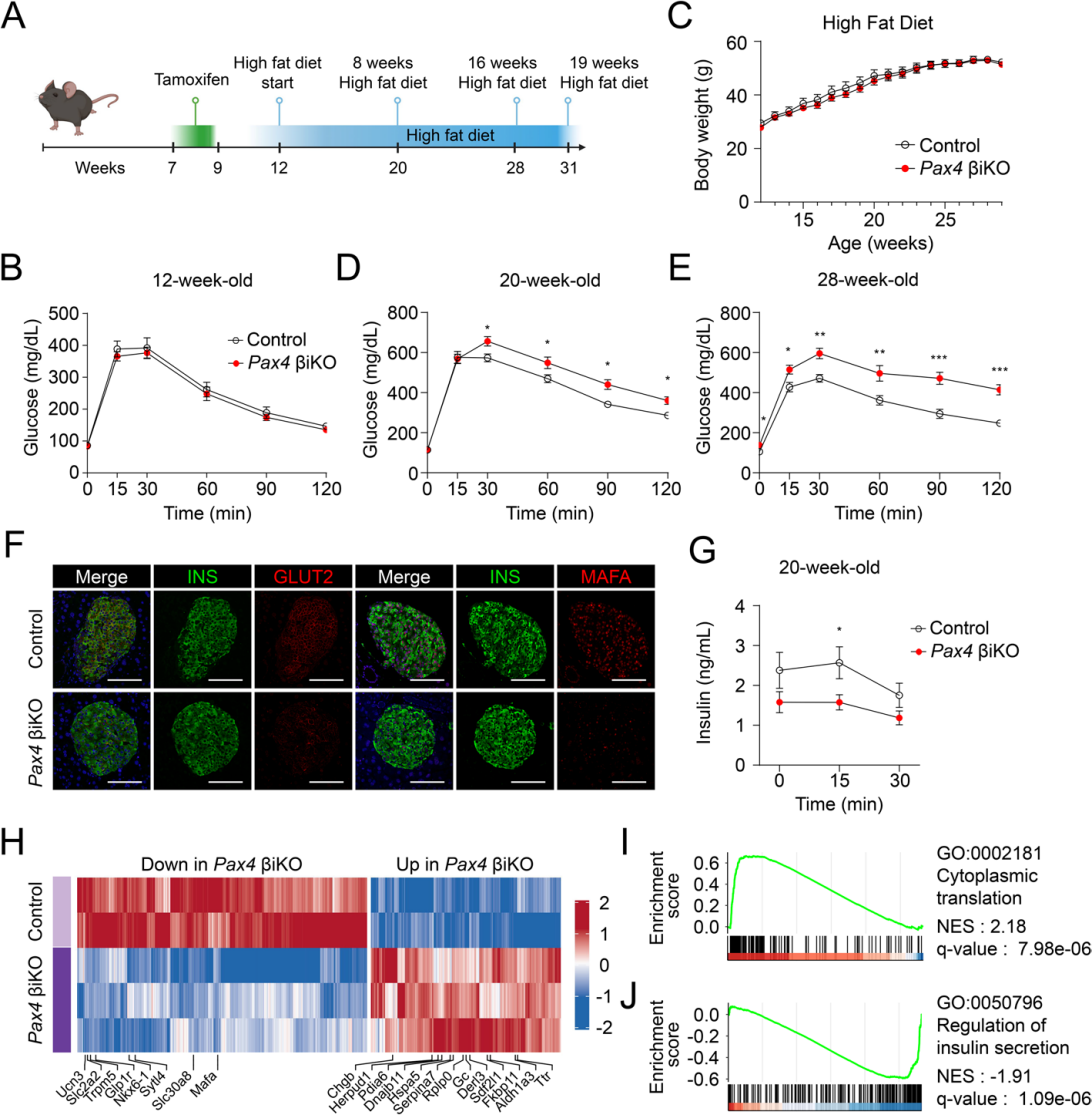
Inducible knockout of *Pax4* in adulthood results in more severe metabolic phenotypes compared to mice carrying the *Pax4* missense mutation. **A** Experimental scheme for *Pax4* βiKO mice. **B** Intraperitoneal glucose tolerance tests after 16-h fasting in *Pax4* βiKO and control mice under standard chow diet (12-week-old); *n* ≥ 4 per group. **C** Body weight of *Pax4* βiKO and control mice fed a high-fat diet; *n* ≥ 4 per group. **D**, **E** Intraperitoneal glucose tolerance tests after 16-h fasting in *Pax4* βiKO and control mice under 8 weeks of high-fat diet (20-week-old) (**D**), and 16 weeks of high-fat diet (24-week-old) (**E**); *n* ≥ 4 per group. **F** Immunofluorescence staining of β cell markers (GLUT2 and MAFA; red) and insulin (green) in pancreas of mice (27-week-old) fed a high-fat diet for 19 weeks. Nuclei are stained with DAPI (blue); *n* = 3 per group. **G** In vivo glucose-stimulated insulin secretion test in control and *Pax4* βiKO mice after 8 weeks of a high-fat diet (20-week-old) after 16-h fasting; *n* = 4–6 per group. **H** Heatmap of differentially expressed genes in islets from control and *Pax4* βiKO mice after 8 weeks of high-fat diet feeding (20-week-old); *n* = 2–3 per group. **I**, **J** GSEA enrichment plots showing enrichment of genes in *Pax4* βiKO islets (compared to control islets) for gene sets termed ‘Cytoplasmic translation (GO:0002181)’ (**I**) and ‘Regulation of insulin secretion (GO:0050796)’ **J**. *P < 0.05; **P < 0.01; ***P < 0.001

We demonstrated that *Pax4* R192H mice do not exhibit developmental defect in pancreatic islet but exhibit impaired β cell compensatory capacity under metabolic stress, accompanied by loss of β cell identity and activation of ER stress. To further validate the role of PAX4 in adult β cell, we generated inducible β cell specific *Pax4* KO (*Pax4* βiKO) mice by crossing *Pax4*^floxed/floxed^ mice with *Pdx1-Cre*^*ERT2*^ mice. Tamoxifen was administered to both control and *Pax4* βiKO mice at 7 to 9 weeks of age, and an HFD was introduced starting at 12 weeks of age (Fig. 5A). Deletion of *Pax4* in adult β cells was confirmed by PCR analysis of genomic DNA and quantitative RT-PCR analysis of *Pax4* mRNA in isolated islets from control and *Pax4* βiKO islets (Supplementary Fig. 8).

At 12 weeks of age, no significant differences were observed in body weight, glucose tolerance, and insulin secretory function between control and *Pax4* βiKO mice fed an SCD (Fig. 5B and Supplementary Fig. 9 A, B). Upon an HFD, body weight remained comparable between the two groups (Fig. 5C). However, *Pax4* βiKO mice developed impaired glucose tolerance after 8 and 16 weeks of HFD (Fig. 5D, E). In immunofluorescence staining, GLUT2-negative/Insulin-positive and MAFA-negative/Insulin-positive β cells were frequently observed in HFD-fed *Pax4* βiKO mice, resulting in decreased GLUT2-positive area and proportion of MAFA-positive cells (Fig. 5F and Supplementary Fig. 10). In contrast, these expression patterns were preserved in *Pax4* R192H mice (Supplementary Fig. 2G). Additionally, *Pax4* βiKO mice on HFD showed a significant reduction in insulin secretion compared with that in controls (Fig. 5G).

To investigate transcriptomic alterations associated with the absence of Pax4 in adult β cells, we performed bulk RNA-seq on islets isolated from *Pax4* βiKO and control mice following 8 weeks of HFD (Fig. 5H–J and Supplementary Fig. 11 A). Key β cell identity genes, such as *Mafa* and *Trpm5*, were significantly downregulated in *Pax4* βiKO islets, while disallowed genes, including *Aldh1a3* and *Serpina7*, were upregulated (Fig. 5H). *Pax4* βiKO islets showed significant upregulation of genes involved in cytoplasmic translation, whereas gene sets related to the regulation of insulin secretion were markedly downregulated (Fig. 5I, J). These transcriptional alterations were similar to those observed in *Pax4* R192H islets, indicating shared transcriptional alterations. Indeed, a significant proportion of DEGs in *Pax4* βiKO islets overlapped with those in *Pax4* R192H islets, with 83 out of 228 genes shared between the two groups (Supplementary Fig. 11B). A strong positive correlation (Pearson’s *R* = 0.65; *P* < 2.2 × 10⁻^1^⁶) was shown between DEGs from bulk RNA-seq of the *Pax4* R192H and *Pax4* βiKO islets upon an HFD (Supplementary Fig. 11 C). Notably, genes co-upregulated in the *Pax4* R192H and *Pax4* βiKO islets, such as *Aldh1a3*, *Serpina7*, *Gc*, and *Ttr,* were enriched in β3 cluster cells in scRNA-seq analysis, which indicates loss of β cell identity. These overlaps in transcriptional changes strongly support the notion that PAX4 is essential for maintaining mature β cell function and adaptation to metabolic stress, with its deficiency leading to impaired glucose tolerance, insulin secretion, and the loss of β cell identity as evidenced by shared transcriptional changes in *Pax4* βiKO and *Pax4* R192H mice under HFD conditions.

### Individuals with the PAX4 R192H variant have a faster decline in β cell function, which is aggravated by obesity

**Fig. 6:**
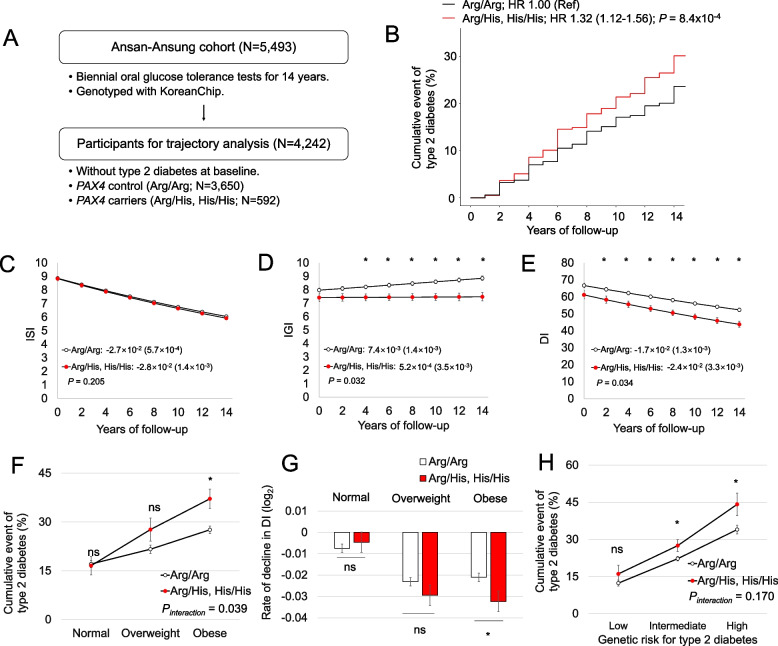
Individuals with the *PAX4* R192H variant have a faster decline in β cell function, which is aggravated by obesity. **A** Schematic diagram of the study protocol. Participants without diabetes at baseline underwent biennial oral glucose tolerance test for 14 years, and their trajectory of β cell function and insulin sensitivity were analyzed based on the *PAX4* R192H variant. **B** Kaplan–Meier plot of cumulative type 2 diabetes incidence. Hazard ratio was calculated with Cox proportional hazards model, adjusted for age, sex and body mass index (BMI). **C**–**E** Trajectory of insulin sensitivity index (ISI), insulinogenic index (IGI), and disposition index (DI) during 14 years of follow-up. Linear-mixed model was applied. The average rate of change per year of log_2_-transformed parameter and its standard error by *PAX4* variant are presented in each panel. *P* value for the difference in the rate of change by the *PAX4* variant is shown. Adjusted for age, sex, and BMI. Test for difference by one-sided t-test. **F** Cumulative event of type 2 diabetes by BMI. BMI below 23 kg/m^2^ were categorized as normal, between 23 kg/m^2^ to 25 kg/m^2^ to overweight and over 25 kg/m^2^ to obese. *P* value for interaction between *PAX4* genotype and BMI for type 2 diabetes incidence is shown, adjusted for age and sex. Test for difference of proportion by one-sided *z*-test. **G** Rate of decline of DI by BMI. Test for difference by one-sided *t*-test. **H** Cumulative event of type 2 diabetes by genetic risk for type 2 diabetes. Individual’s polygenic risk for type 2 diabetes were calculated and stratified into low (1st quintile), intermediate (2nd to 4th quintiles) and high genetic risk (5.^th^ quintile). *P* value for interaction between *PAX4* genotype and type 2 diabetes genetic risk score for type 2 diabetes incidence are shown, adjusted for age, sex, and BMI. Test for difference of proportion by one-sided *z*-test. *P < 0.05

Individuals with *PAX4* R192H variant develop type 2 diabetes at an earlier age and have impaired β cell function compared with those without the variant [5]. To better understand longitudinal dynamics of β cell function in those with the *PAX4* R192H variant, we analyzed the data from the Ansan-Ansung cohort [35, 46], a community-based prospective cohort in South Korea, in which participants underwent biennial 75-g oral glucose tolerance tests (OGTT). With this cohort, we compared the trajectory of OGTT-derived insulinogenic index at 60 min (IGI_60,_ a measure of β cell secretory index), insulin sensitivity index (ISI) and disposition index (DI, β cell function adjusted for insulin sensitivity) for up to 14 years (*n* = 4,242) (Fig. 6A and Supplementary Table 1). Carriers of the *PAX4* R192H had a 1.32-fold increased risk for type 2 diabetes (Fig. 6B). There was no significant difference in changes in insulin sensitivity over time; however, rate of decline in β cell function was accelerated in those carrying the *PAX4* R192H variant (Fig. 6C–E). At baseline, IGI_60_ was not different between the two groups; however, by year four, a significant difference emerged (Arg/Arg vs Arg/His, His/His: *P* = 0.025), which continued to widen during subsequent follow-ups (Fig. 6D). The DI was significantly lower in the carrier group starting from year two (*P* = 0.033), and its rate of decline was significantly faster in carriers compared with that in wildtype individuals (1.4-fold faster decline in log_2_-transformed DI, *P* = 0.034) (Fig. 6E).

We next investigated whether obesity aggravates β cell dysfunction in individuals carrying the *PAX4* R192H variant, as observed in our HFD-fed mouse models. Participants were categorized into normal, overweight, and obese groups based on BMI. While no significant difference in type 2 diabetes risk was observed between wildtype individuals and carriers in the normal BMI group, the risk gradually diverged in the overweight group and became significantly higher among carriers in the obese group (Fig. 6F, *P*_*interaction*_ = 0.039). Consistently, the rate of decline in the DI was significantly steeper in the obese group, whereas no significant difference was observed in the other BMI categories (Fig. 6G).

Given that obesity is a modifiable risk factor, we further examined whether genetic predisposition to type 2 diabetes interacts with the *PAX4* variant. To do this, we constructed a genetic risk score independent of the variant of our interest (± 500 kb apart, linkage disequilibrium *r*^2^ < 0.05). Although the interaction genetic risk score and *PAX4* variant did not reach statistical significance, a greater divergence of type 2 diabetes incidence was observed in the intermediate and high genetic risk groups (Fig. 6H). Overall, these findings suggest that individuals with the PAX4 variant have reduced β cell compensatory capacity in response to declining insulin sensitivity over time, and this impairment in compensatory capacity is further exacerbated by obesity and genetic predisposition to type 2 diabetes.

## Discussion

The *PAX4* R192H variant is the only functional variant identified with genome-wide significance for type 2 diabetes in large-scale exome sequencing studies, with a substantial effect size and strong East Asian specificity. However, its functional and mechanistic role in pathogenesis of type 2 diabetes has remained unclear. In this study, we sought to elucidate the metabolic impact of this variant by integrating human genetic and metabolic phenotype data with in vivo experimental model of *PAX4* R192H variant. Specifically, we demonstrated an accelerated decline in β cell function among carriers in a large community-based prospective human cohort and independently confirmed impaired β cell compensatory capacity in *Pax4* R192H knock-in mice under HFD conditions.

By leveraging a *Pax4* R192H knock-in and βiKO mice model, we investigated the mechanistic basis by which this East Asian-specific variant impairs β cell adaptation to metabolic stress. *Pax4* R192H knock-in mice exhibited normal pancreatic endocrine development and had similar body weight, glucose tolerance, insulin sensitivity, or β cell area under a SCD compared to wildtype mice. However, under HFD-induced metabolic stress, *Pax4* R192H mice displayed impaired glucose tolerance, reduced insulin secretion, similar insulin sensitivity and diminished β cell area expansion. Transcriptomic analysis of islets revealed minimal differences under SCD but significant changes under HFD, with upregulated ER stress and cytoplasmic translation pathways and downregulated β cell identity genes (e.g., *Mafa* and *Slc2a2*) in *Pax4* R192H mice. Notably, a β cell cluster (β3) characterized by high translation process activity and reduced β cell maturity was enriched in *Pax4* R192H islets under HFD condition, displaying increased ER stress, ribosomal gene expression, and dedifferentiation marker genes, indicative of a stress-vulnerable phenotype characterized by loss of β cell identity. Comparison with *Pax4* βiKO mice revealed a strong correlation in differentially expressed genes (Pearson’s *R* = 0.65), with βiKO mice showing more severe phenotypic defects. Immunofluorescence analysis revealed a marked reduction in β cell markers MAFA and GLUT2 in *Pax4* βiKO islets, a phenotype not observed in *Pax4* R192H islets. Furthermore, while prolonged HFD feeding did not worsen glucose tolerance deficits in *Pax4* R192H mice, *Pax4* βiKO mice exhibited progressively impaired glucose tolerance with extended HFD exposure, indicating greater susceptibility to metabolic stress. Finally, in a prospective human cohort, *PAX4* R192H carriers showed a faster decline in β cell function (1.4-fold faster decline in disposition index, *P* = 0.034), driven by impaired insulin secretion, with increasing BMI further exacerbating their risk for type 2 diabetes (*P*_*interaction*_ = 0.039). These findings demonstrate that the *PAX4* R192H variant impairs β cell adaptation to metabolic stress, thereby contributing to the increased risk of type 2 diabetes.

The results from our study support three notable conclusions. First, our findings suggest that the *PAX4* R192H variant represents an important example of genetic heterogeneity in type 2 diabetes contributing to phenotypic differences between populations. Previous epidemiological studies have highlighted that individuals with type 2 diabetes in the East Asian population tend to develop the disease at lower BMI, with a predominant β cell dysfunction phenotype compared with those in the European population [35, 47]. The *PAX4* R192H variant is exclusive but relatively common in the East Asian population (minor allele frequency 10%) [48]. We demonstrated an accelerated decline in β cell function among *PAX4* R192H carriers in a prospective human cohort and independently confirmed impaired β cell compensatory capacity in *Pax*4 R192H mice under HFD, highlighting consistent evidence across both human and experimental models. These findings provide a mechanistic explanation for the increased vulnerability to β cell failure observed in East Asian populations. Collectively, our results suggest that population-specific genetic architecture, such as the *PAX4* R192H variant, may underlie part of the ethnic heterogeneity observed in type 2 diabetes pathophysiology.

Second, our findings demonstrate that metabolic stress exacerbates β cell dysfunction in the presence of the *PAX4* R192H variant, suggesting the importance of lifestyle modification in individuals carrying the variant. Several studies suggest that Pax4 expression may be transcriptionally regulated in response to metabolic stress. In particular, HFD feeding has been shown to induce Pax4 expression via the SREBP1c-PAX4 axis, linking lipid-sensitive transcriptional programs to β cell compensation [12]. In addition, Pdx1 and mitogenic signaling pathways, including GLP-1 and activin A, have been implicated as upstream regulators of Pax4, suggesting that Pax4 expression is dynamically modulated by metabolic and growth factor-dependent cues [49–51]. In *Pax4* R192H mice, HFD-induced insulin resistance aggravated glucose intolerance and impaired β cell area expansion. Similarly, in humans, obesity accelerated the decline in β cell function and increased type 2 diabetes risk among *PAX4* R192H carriers. These results suggest that lifestyle modification should be emphasized in individuals carrying the variant. Recent advances in incretin-based therapies, particularly GLP-1 receptor agonists, have shown robust weight-lowering effects [52, 53], which may ameliorate insulin resistance and consequently reduce metabolic stress on β cells in *PAX4* R192H carriers. Moreover, previous studies have shown that GLP-1 increases PAX4 expression in human islets [50], therefore, GLP-1 receptor agonists may partially compensate for the reduced PAX4 activity caused by the R192H variant. Further studies are warranted to explore whether GLP-1 based therapies could decelerate β cell function decline in individuals with the *PAX4* R192H variant.

Third, by using multiple genetically modified mouse models, we were able to delineate the adult-specific role of PAX4 and distinguish the effects of the PAX4 R192H variant from those of PAX4 KO. Previous studies have suggested that PAX4 missense variants compromise β cell resilience to stress. For example, the p.Arg121Trp variant (R121W in human; R129W in mouse), originally reported as a type 2 diabetes risk allele in early candidate gene studies [54, 55], was shown to abolish the protective effect of PAX4 against cytokine- or streptozotocin-induced stress in overexpression systems [13], consistent with a loss-of-function effect of this mutation. Several functional and structural studies have suggested that the R192H variant, located within the DNA-binding domain of PAX4, reduces the transcriptional activity of β cell-associated target genes, such as INS [15, 16, 56]. However, it remained unclear whether PAX4 R192H primarily affects pancreatic endocrine specification or the maintenance of compensatory function under metabolic stress. In a recent study by Lau et al., PAX4 knockdown or introduction of the R192H variant in the human β cell line EndoC-βH1 impaired insulin secretion and altered β cell gene expression, indicating a functional role for PAX4 in mature β cells [16]. Meanwhile, donor-derived hiPSCs carrying the R192H variant and the p. Tyr186X variant exhibited impaired endocrine differentiation, while PAX4 deletion in hiPSCs led to derepression of α cell gene programs and increased formation of polyhormonal endocrine cells [16]. These findings suggest that PAX4 R192H may disrupt pancreatic endocrine specification during development. In our study, *Pax4* R192H knock-in mice did not display overt abnormalities in islet architecture, endocrine cell composition, or transcriptional signatures during embryonic development. Metabolic phenotypes were likewise comparable to those of wildtype controls under SCD, indicating that the R192H variant alone is insufficient to perturb pancreatic endocrine development in vivo under basal conditions. To exclude the possibility that subtle developmental defects in *Pax4* R192H mice account for impaired adaptive responses, we generated β cell-specific inducible *Pax4* βiKO mice. Upon HFD, *Pax4* βiKO mice exhibited defective insulin secretion and impaired transcriptional adaptation, confirming a key role for PAX4 in maintaining β cell function under metabolic stress. In line with its broader role in endocrine lineage plasticity, ectopic expression of Pax4 in mature α cells has been shown to activate β cell transcriptional programs and induce α-to-β cell transdifferentiation [57]. Together, these data support the notion that PAX4 is not only essential during development but also required for the maintenance and adaptive capacity of mature β cells. While the loss-of-function variant, PAX4 R192H, compromises β cell adaptation against metabolic stress, its precise role in early pancreatic endocrine specification warrants further investigation.

Consistent with impaired mature β cell identity, we observed upregulation of disallowed and dedifferentiation-associated genes, including *Aldh1a3*, *Gc*, and *Ttr*, in both *Pax4* R192H and β cell-specific *Pax4* knockout islets under HFD conditions. *Aldh1a3* and *Gc* have been reported as markers of β cell dedifferentiation, while *Gc* and *Ttr* are also enriched in α cell transcriptional signatures [41–44]. Analysis of published PAX4 CUT&RUN datasets did not reveal direct PAX4 binding at these loci, suggesting that these genes are unlikely to be direct targets of PAX4 [45]. Instead, these changes likely reflect secondary consequences of impaired β cell identity and adaptive capacity under metabolic stress, consistent with observations in other models of β cell identity loss [58].

Comparison of transcriptomic changes observed in *Pax4* R192H and *Pax4* βiKO islets showed substantial difference, with more DEGs detected in *Pax4* R192H islets. These findings suggest that the functional consequences of the *PAX4* R192H variant are not fully recapitulated by loss of Pax4 in adult β cells. The difference may, at least in part, reflect the timing of Pax4 perturbation, as *Pax4* R192H captures both developmental and adult-stage consequences of impaired Pax4 function, whereas the *Pax4* βiKO model reflects only the adult-stage requirement for Pax4. In addition, the *Pax4* R192H variant may retain partial chromatin binding while exhibiting impaired transcriptional competence, potentially interfering with transcriptional complexes or stress-adaptive gene programs in a manner not recapitulated by Pax4 deletion. By contrast, inducible deletion of Pax4 in adult β cells may allow partial compensation by other transcription factors within the β cell regulatory network, thereby limiting the extent of transcriptomic disruption.

## Conclusion

In summary, our study provides functional mechanism of the type 2 diabetes-associated *PAX4* R192H variant in the disease pathogenesis. The *Pax4* R192H mice displayed impaired β cell compensatory capacity under metabolic stress, with single cell transcriptomic analyses demonstrating enhanced ER stress and reduced β cell maturity. In a community-based prospective cohort, carriers of the *PAX4* R192H exhibited a faster decline in β cell function, with increasing BMI further exacerbating their risk of developing type 2 diabetes. Our results provide mechanistic role of the *PAX4* R192H variant in type 2 diabetes pathogenesis and highlight the importance of lifestyle modification for diabetes prevention in carriers of this variant.

## Supplementary Information

Supplementary Material 1.

## Data Availability

The sequencing data (scRNA-seq and bulk RNA-seq) generated during this study have been deposited in the Gene Expression Omnibus (GEO) repository under accession number GSE330587, GSE330588. All other data generated or analyzed during this study are included in this published article and its supplementary information files.

## Abbreviations

Pax4: Paired box gene 4
KO: Knockout
HFD: High-fat diet
GSIS: Glucose-stimulated insulin secretion
scRNA-seq: Single-cell RNA sequencing
FACS: Fluorescence-activated cell sorting
IF: Immunofluorescence
DEGs: Differentially expressed genes
BMI: Body mass index
