# Supplementary material for: PAX4 R192H variant impairs β cell function by disrupting β cell identity and compensatory capacity in response to metabolic stress
Source: Genome Med. 2026 May 28;18:111. doi: 10.1186/s13073-026-01669-z (PMC13403659; doi:10.1186/s13073-026-01669-z)
Supplement: Supplementary file 1 — Supplementary Material 1. [file 13073_2026_1669_MOESM1_ESM.docx]

# SUPPLEMENTARY DATA

**Supplementary Table**

**Supplementary Table 1. Baseline characteristics of study participants by *PAX4* R192H genotype.**

| ***PAX4* R192H genotype** | ***PAX4* Arg/Arg**  **(n=3,650)** | ***PAX4* Arg/His, His/His**  **(n=592)** | ***P* value^*^** |
| --- | --- | --- | --- |
| **Age (years)** | 51.1 (8.4) | 50.9 (8.3) | 0.676 |
| **Sex (%, male)** | 1703 (47) | 291 (49) | 0.278 |
| **Body mass index (kg/m^2^)** | 24.4 (3.0) | 24.7 (3.0) | 0.014 |
| **Fasting plasma glucose (mg/dL)** | 82.8 (8.4) | 83.8 (8.6) | 0.004 |
| **2 h glucose (mg/dL)** | 113 (29) | 116 (31) | 0.054 |
| **HbA1c (%)** | 5.52 (0.34) | 5.56 (0.35) | 0.017 |
| **Fasting insulin (pmol/L)^†^** | 6.3 (1.9) | 6.6 (1.7) | 0.080 |
| **IGI_60_^†^** | 6.8 (3.5) | 6.3 (3.5) | 0.167 |
| **ISI^†^** | 9.8 (1.8) | 9.5 (1.8) | 0.168 |
| **Disposition Index^†^** | 70.9 (3.4) | 61.1 (3.2) | 0.021 |
| **Total Cholesterol (mmol/L)^†^** | 187 (1.2) | 188 (1.2) | 0.774 |
| **Triglycerides (mmol/L)^†^** | 134 (1.6) | 138 (1.6) | 0.270 |
| **HDL cholesterol (mmol/L)^†^** | 44.1 (1.2) | 44.3 (1.2) | 0.555 |
| **LDL cholesterol (mmol/L)^†^** | 111 (1.3) | 110 (1.4) | 0.432 |

Data are unadjusted means (standard deviation), geometric means (geometric standard deviation), or n (%).

**P* values for the difference between genetic risk groups are calculated with *t*-test and the χ² test for continuous and categorical variables, respectively.

^†^Variables were log-transformed before statistical analysis and shown as geometric mean (geometric standard deviation).

IGI_60_, insulinogenic index at 60 min; ISI, insulin sensitivity index.

**Supplementary Figure**

**
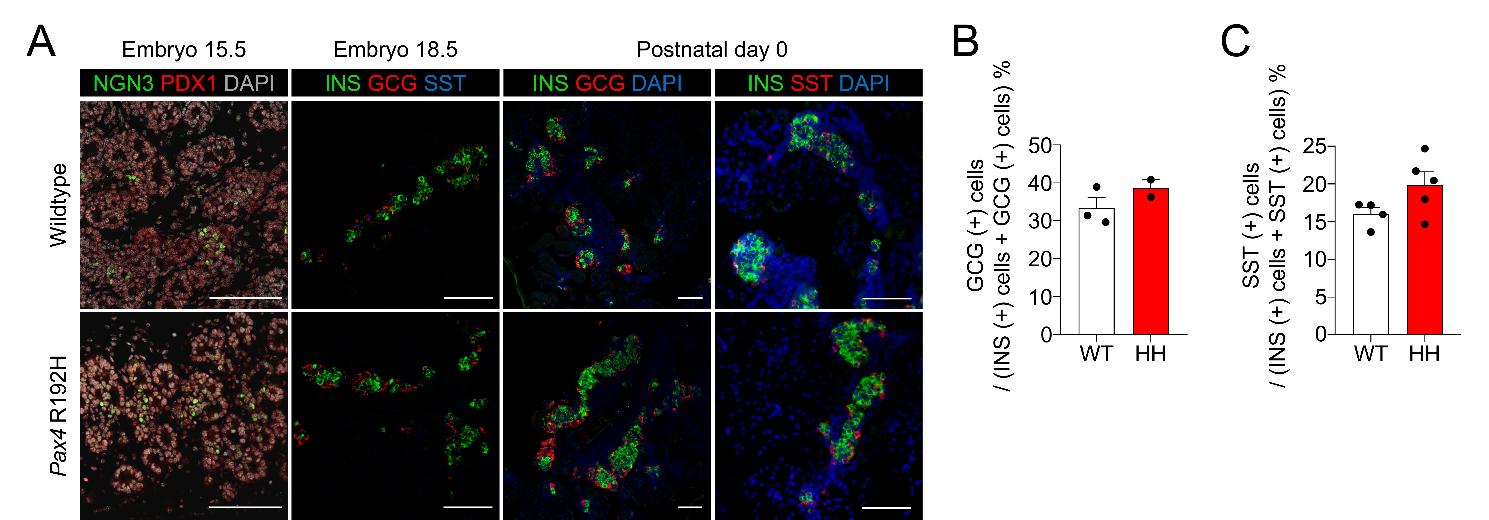
**

**Supplementary Figure 1. Pancreatic endocrine development was not altered in *Pax4* R192H mice**

(A) Immunofluorescence staining of Neurogenin3 (green) and Pdx1 (red) in the pancreas of wildtype and *Pax4* R192H mice at embryonic day 15.5 (E15.5), with nuclei stained with DAPI (gray). Immunofluorescence staining of endocrine hormones, including glucagon(red) and somatostatin(blue), and insulin (green) in the pancreas of wildtype and *Pax4* R192H mice at embryonic day 18.5 (E18.5). Immunofluorescence staining of endocrine hormones in the pancreas at postnatal day 0 (P0), showing Insulin (green), glucagon (red) or somatostatin (red) with nuclei stained with DAPI (blue); n ≥3 per group. Scale bar, 100 μm. (B,C) The number of glucagon positive (GCG⁺) α cells (B) and somatostatin-positive (SST⁺) δ cells (C) was quantified in pancreatic sections from wildtype (white bars) and *Pax4* R192H (red bars) mice at postnatal day 0; n ≥2 per group.


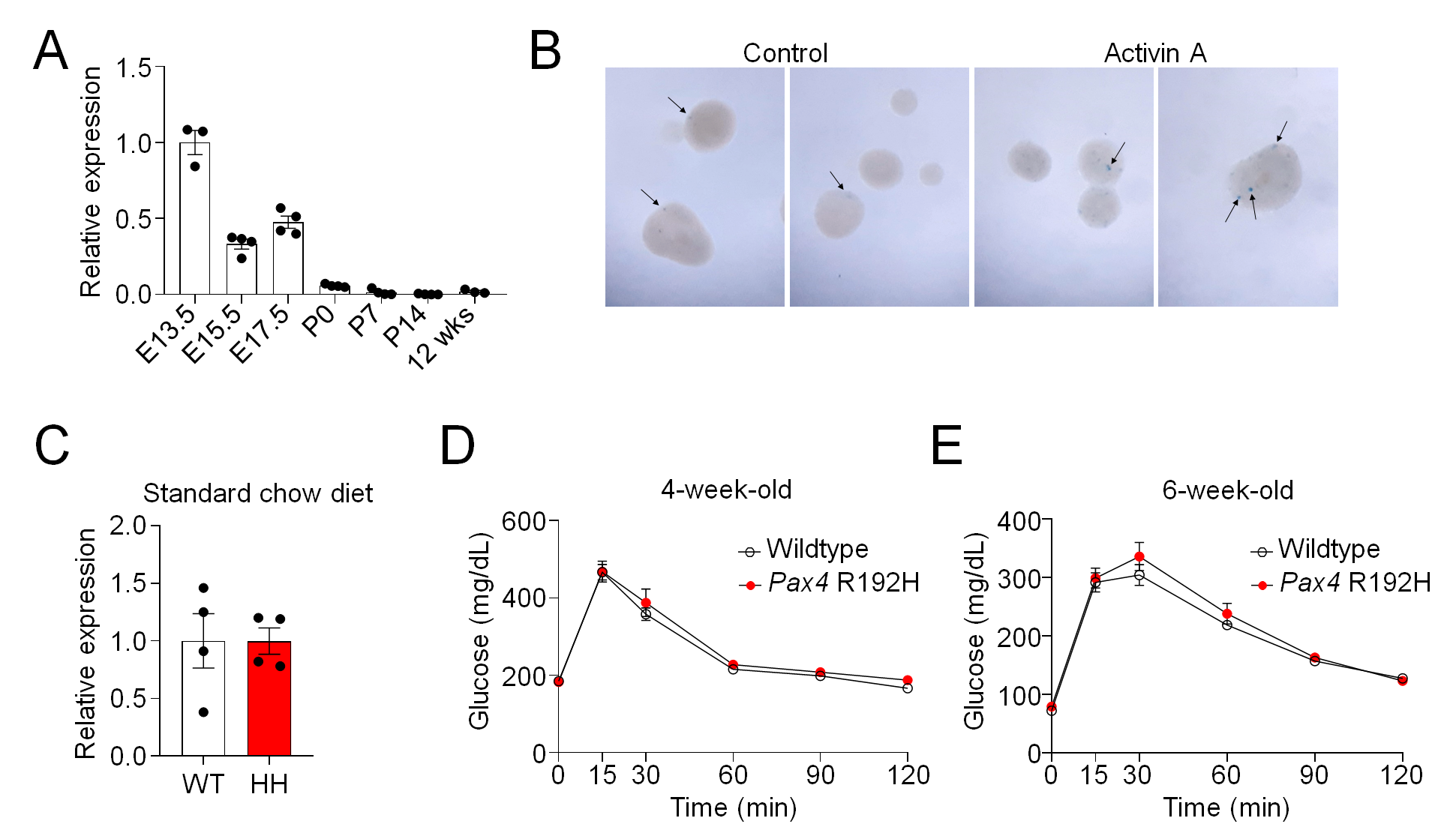


**Supplementary Figure 2. Pax4 expression in adult islets and glucose tolerance of *Pax4* R192H mice**

(A) *Pax4* mRNA expression in whole pancreas at embryonic day 13.5 (E13.5) through postnatal day 14 (P14), and in isolated pancreatic islets from 12-week-old adult mice, as measured by quantitative RT–PCR. The accompanying table shows the mean Ct values obtained from qRT–PCR for each condition, providing information on transcript abundance. Expression levels were normalized to *Actb* as an internal control, which exhibited stable Ct values of approximately 21 across all samples, supporting the reliability of the qRT–PCR measurements. (B) Representative bright-field images of isolated pancreatic islets from *Pax4^LacZ/+^* reporter mice stained with X-gal. X-gal-positive cells (arrows) were detected in adult islets, indicating Pax4 expression with heterogeneous distribution among islet cells. (C) *Pax4* mRNA expression in isolated islets from *Pax4* R192H mice under standard chow diet conditions. The accompanying table shows the mean Ct values obtained from qRT–PCR for each condition, providing information on transcript abundance. *Actb* was used as an endogenous control. n ≥ 3 mice per group. (D,E) Intraperitoneal glucose tolerance test in wildtype and *Pax4* R192H mice after 16-hour fasting in 4-week-old (D) and 6-week-old (E); n ≥5 per group. WT, wildtype; HH, *Pax4* R192H; GCG, glucagon; SST, somatostatin; INS, insulin.


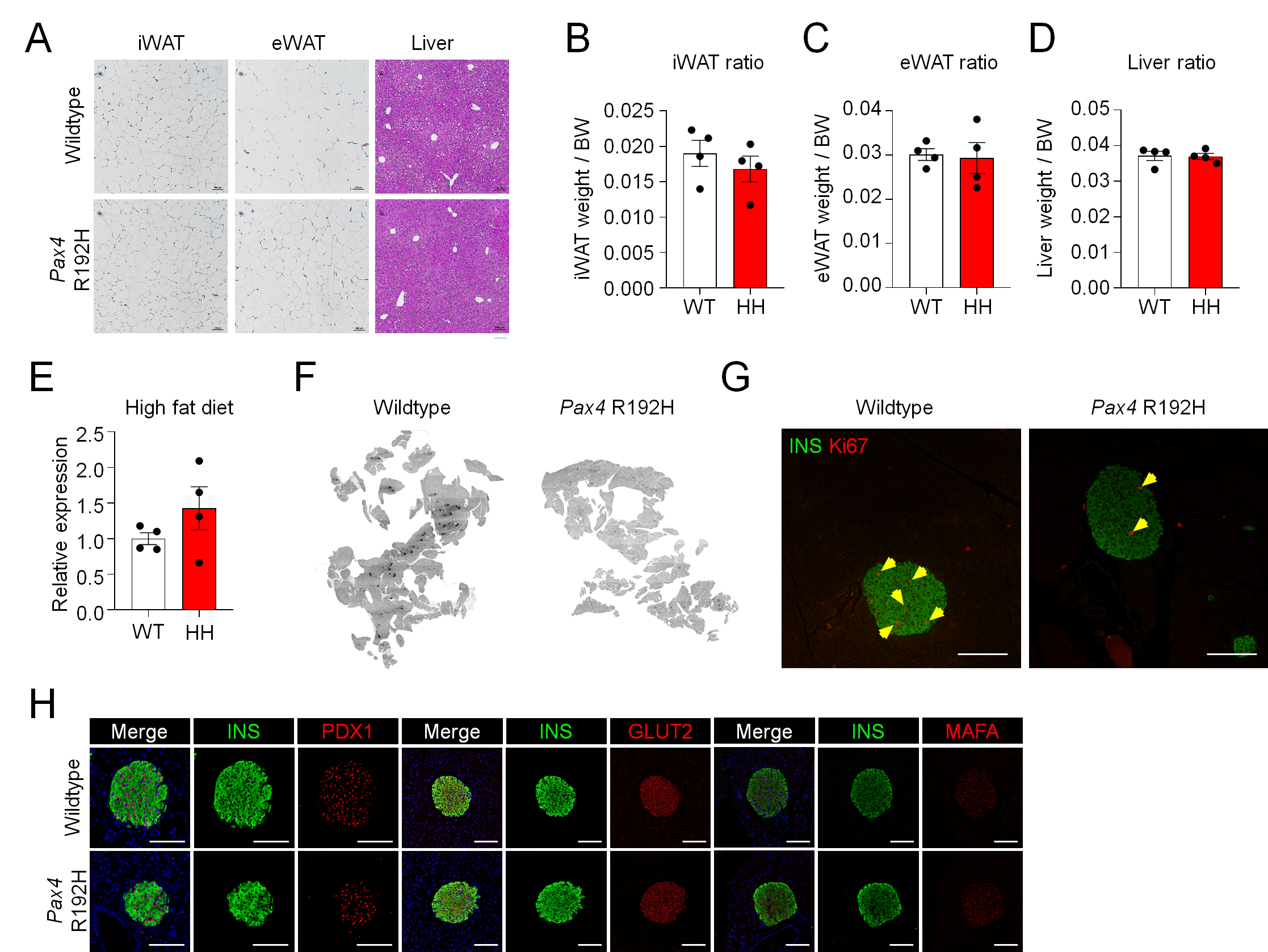


**Supplementary Figure 3. Metabolic phenotypes of *Pax4* R192H mice under high fat diet**

(A) Representative hematoxylin and eosin (H&E) staining of adipose tissue (iWAT, eWAT) and liver sections from mice fed a high fat diet for 4 weeks (16-week-old); n=4 per group. (B-D) Adipose tissue and liver to body weight ratio of iWAT (B), eWAT (C), and liver (D); n=4 per group. (E) *Pax4* mRNA expression in isolated islets from *Pax4* R192H mice under high fat diet conditions. The accompanying table shows the mean Ct values obtained from qRT–PCR for each condition, providing information on transcript abundance. *Actb* was used as an endogenous control. n ≥ 3 mice per group. (F) Immunohistochemical staining of insulin in pancreatic sections from wildtype and *Pax4* R192H mice fed a high fat diet for 4 weeks (16-week-old); n=5 per group. (G) Immunofluorescence staining with Ki67 (red) and insulin (green) of wildtype and *Pax4* R192H mice fed 2 weeks of high fat diet (14-week-old). Yellow arrows=Ki67 and insulin co-positive cells; n=4 per group. Scale bar, 100 μm. (H) Immunofluorescence of β cell markers (PDX1, UCN3 and MAFA) and insulin in pancreatic sections from mice fed 4 weeks of high fat diet (16-week-old); n=4 per group. Scale bar, 100 μm. BW, body weight; WT, wildtype; HH, *Pax4* R192H.

**
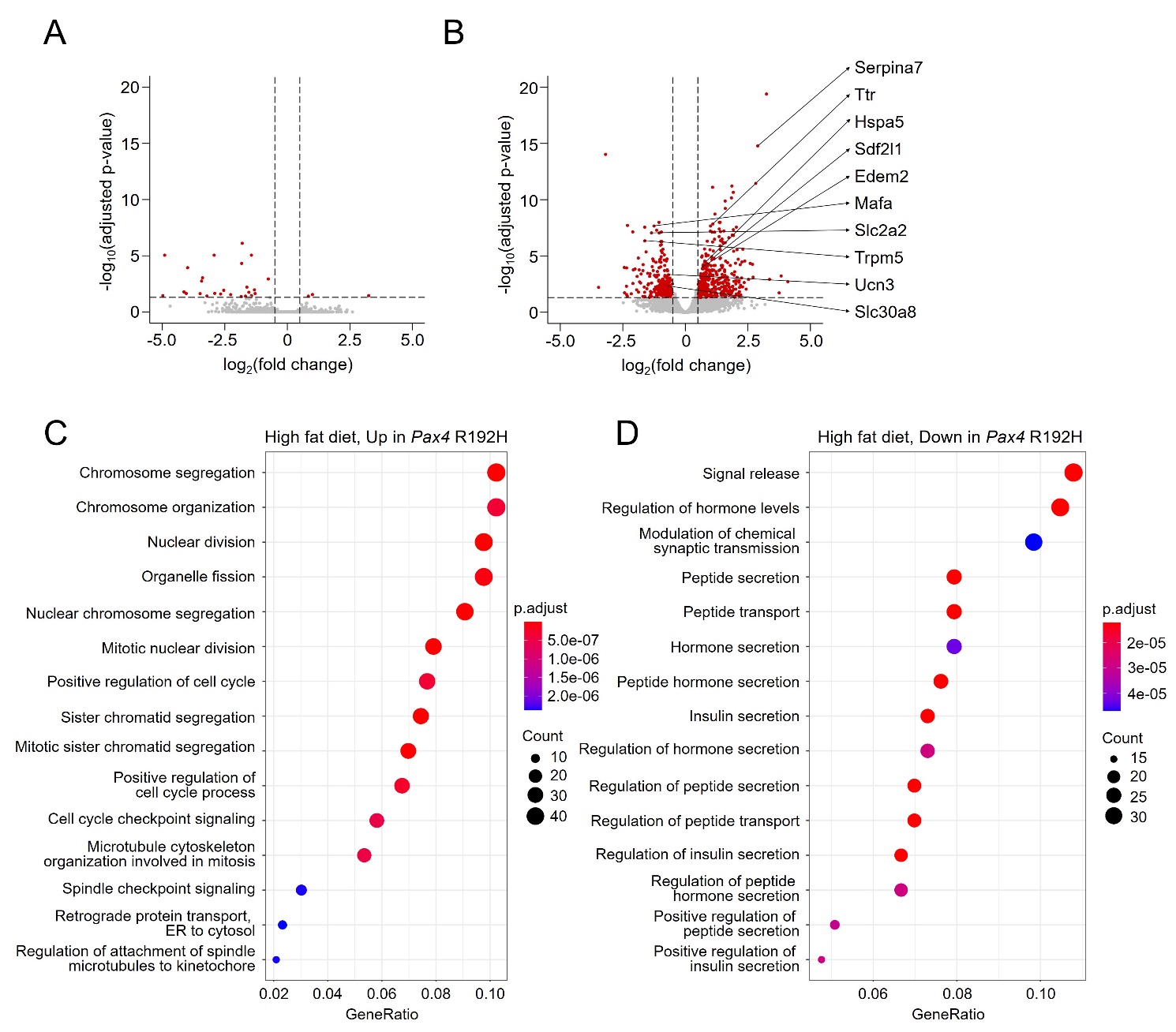
**

**Supplementary Figure 4. Gene set enrichment analysis reveals altered transcriptional programs in *Pax4* R192H islets under high fat diet**

(A, B) Volcano plots showing differential gene expression between wildtype and *Pax4* R192H mice within each diet condition: (A) SCD at 12 weeks; (B) HFD at 16 weeks. Vertical lines indicate |log_2_(fold change)| = 0.5, and horizontal line indicates -log_10_(adjusted *P* value) = 1.3. (C, D) Results of over-representation analysis showing gene sets in the GO Biological Process pathway upregulated (C) or downregulated (D) in *Pax4* R192H islets under high fat diet (16-week-old).


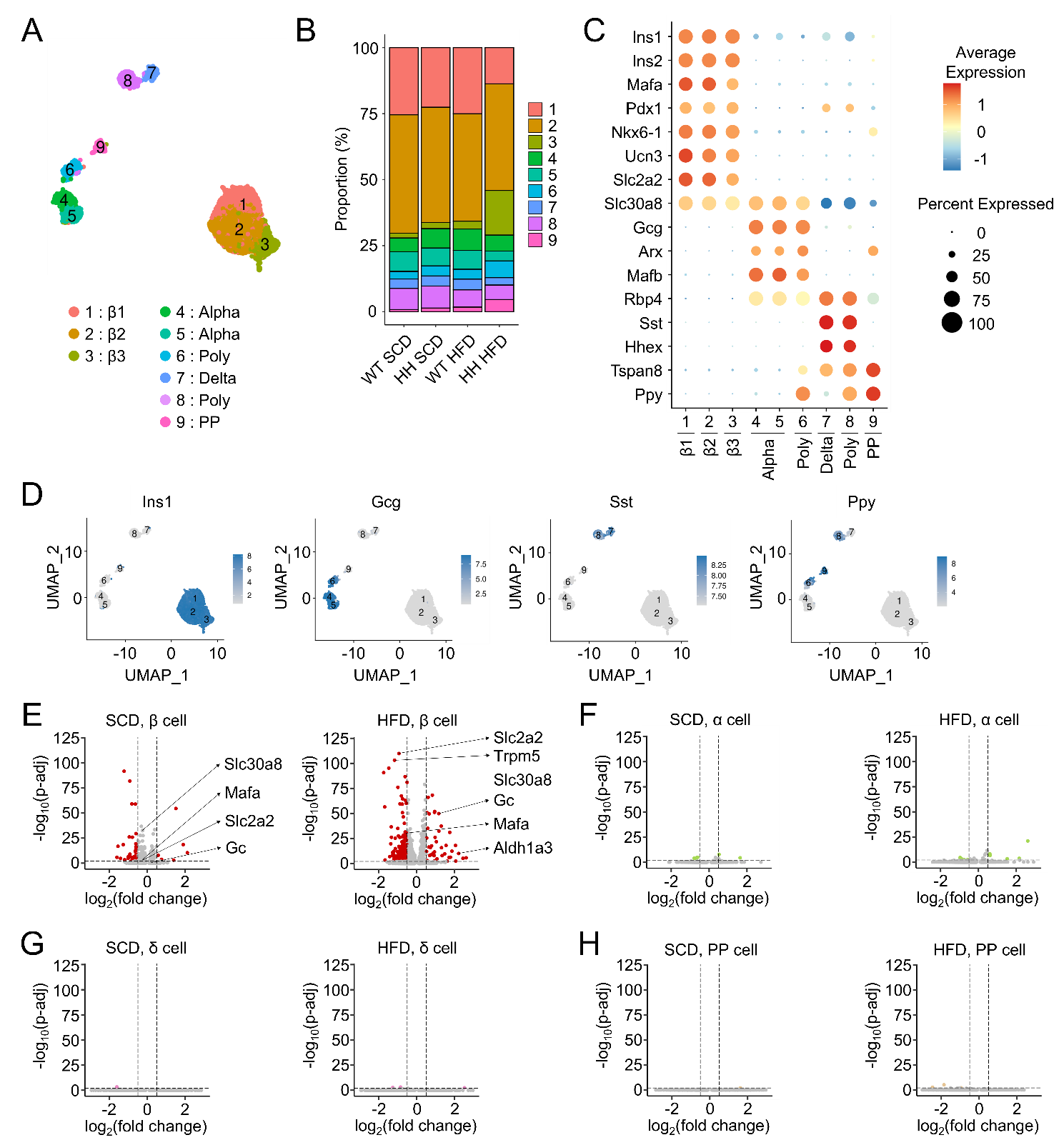


**Supplementary Figure 5. *Pax4* variant induces distinct transcriptomic changes in β cells compared to other endocrine cells**

(A) UMAP plot of pancreatic endocrine cells from wildtype and *Pax4* R192H mice fed standard chow diet (12-week-old) or high fat diet (16-week-old). Colors represent a distinct cell cluster. (B) Composition pattern of cell clusters in wildtype and *Pax4* R192H mice fed standard chow diet (12-week-old) or high fat diet (16-week-old). (C) Dot plot showing relative expression of endocrine cell markers in each cluster. Colors indicate relative expression levels and dot sizes indicate percentage of cells expressing each gene. (D) UMAP plots showing the scaled expression of representative islet hormone genes (*Ins1*, *Gcg*, *Sst*, and *Ppy*) (E–H) Volcano plots showing differential gene expression in β cells (E), α cells (F), δ cells (G) or PP cells (H) between wildtype and *Pax4* R192H under standard chow diet or high fat diet. Vertical lines indicate |log_2_(fold change)| = 0.5, and horizontal line indicates -log_10_(p-adj) = 2. WT SCD, wildtype with standard chow diet; HH SCD, *Pax4* R192H homozygous knock-in with standard chow diet; WT HFD, wildtype with high fat diet; HH HFD, *Pax4* R192H homozygous knock-in with high fat diet.

**
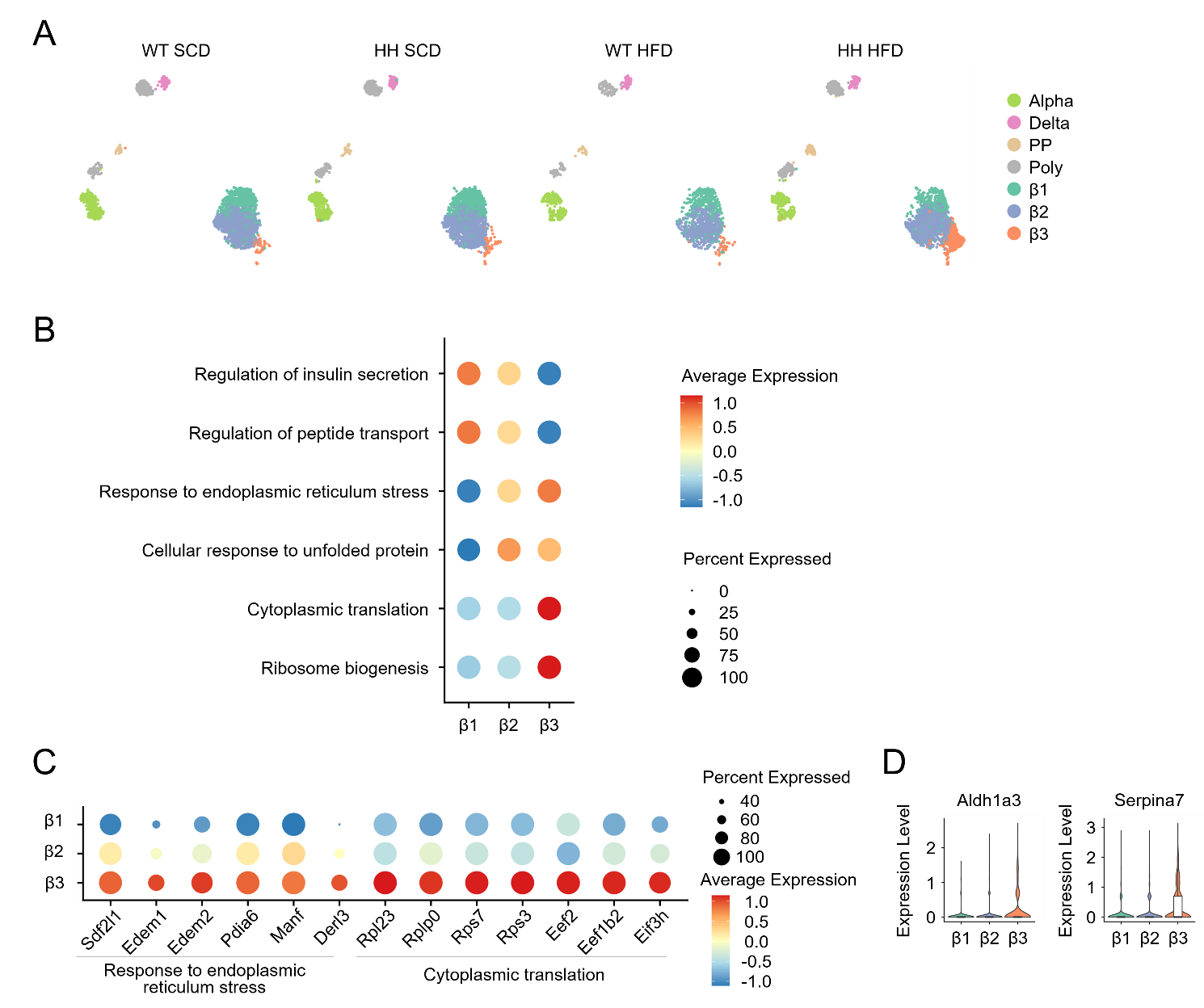
**

**Supplementary Figure 6. *Pax4* variant induces loss of β cell identity with activation of UPR and translation process**

(A) UMAP plot showing pancreatic endocrine cells from wildtype and *Pax4* R192H mice fed standard chow diet (12-week-old) or high fat diet (16-week-old). Colors represent cell types. (B) Dot plot showing expression levels of genes included in Gene Ontology (GO) terms identified by pathway analysis of β cell clusters. Colors indicate relative expression levels and dot sizes indicate percentage of cells expressing genes included in gene sets. (C) Dot plot showing relative expression of genes in gene sets termed 'Response to endoplasmic reticulum stress’ and ‘Cytoplasmic translation’ in β cell clusters. Colors indicate relative expression levels and dot sizes indicate percentage of cells expressing each gene. (D) Violin plot showing expression levels of *Aldh1a3 and Serpina7* among β cell clusters. WT SCD, wildtype with standard chow diet; HH SCD, *Pax4* R192H homozygous knock-in with standard chow diet; WT HFD, wildtype with high fat diet; HH HFD, *Pax4* R192H homozygous knock-in with high fat diet.


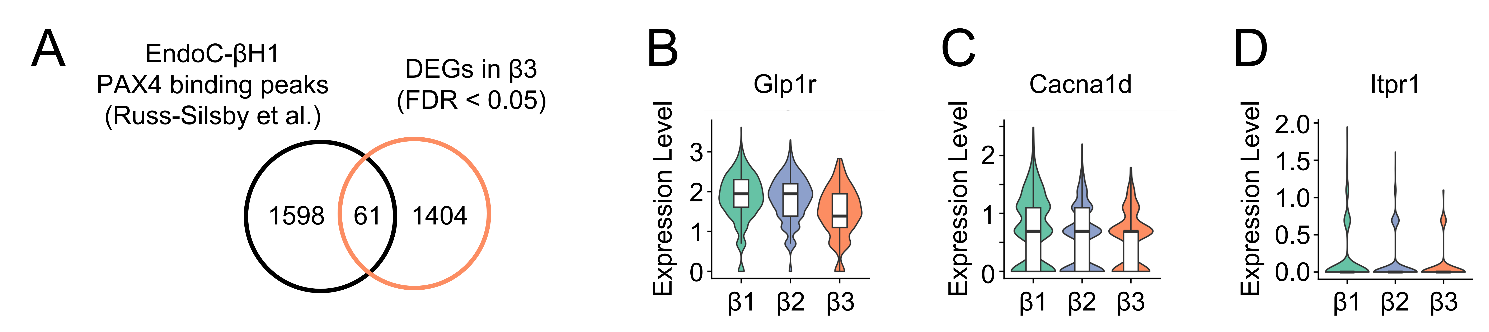


**Supplementary Figure 7. PAX4 binding targets are associated with β cell function**

(A) Venn diagram showing the number of genes associated with PAX4 binding peaks in EndoC-βH1 (Russ-Silsby et al. Mol Metab. 2025) and differentially expressed genes in β3 cluster from the scRNA-seq analysis in Figure 4. (B-D) Violin plots showing expression levels of *Glp1r* (B), *Cacna1d* (C), and *Itpr1* (D) among β cell clusters from wildtype and *Pax4* R192H mice fed high fat diet.

**
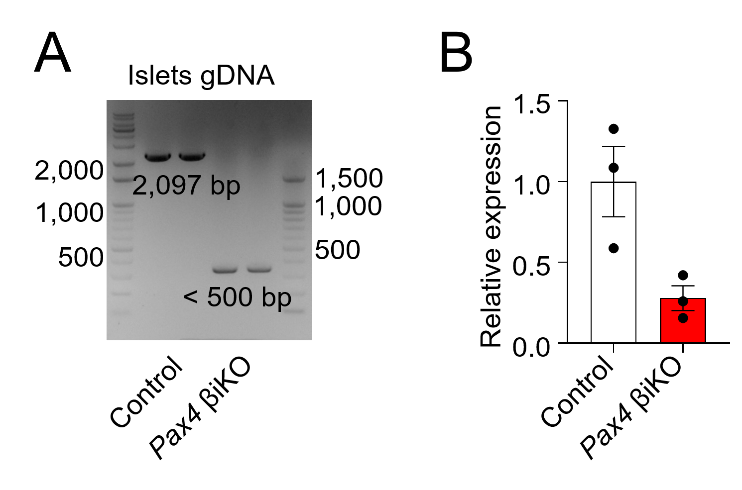
**

**Supplementary Figure 8*.* Pax4 Deletion in *Pax4* βiKO Islets**

(A) PCR analysis of genomic DNA purified from pancreatic islets confirming Cre-mediated recombination of the Pax4 floxed allele in *Pax4* βiKO mice. The intact floxed allele yields a 2,097 bp PCR product, whereas recombined alleles generate a <500 bp fragment. (B) Quantitative RT–PCR analysis of Pax4 mRNA expression in isolated islets from control and *Pax4* βiKO mice. n=3 mice per group.

**
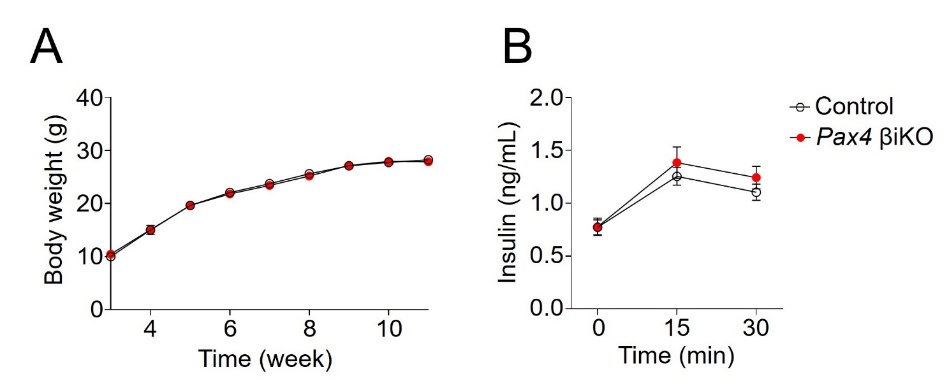
**

**Supplementary Figure 9. *Pax4* βiKO mice exhibit comparable phenotypes under standard chow diet conditions**(A) Body weight of *Pax4* βiKO and control mice fed a standard chow diet (12-week-old); n=8–11 per group. (B) *In vivo* glucose-stimulated insulin secretion test in 12-week-old control and *Pax4* βiKO mice fed a standard chow diet for 16-hour fasting; n=4–7 per group.

**
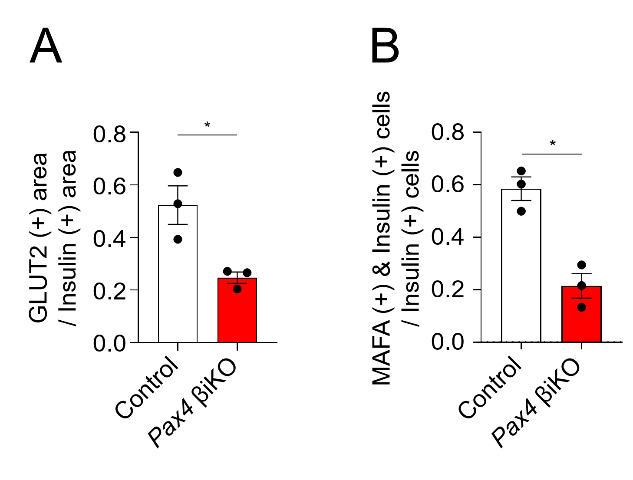
**

**Supplementary Figure 10. Decreased GLUT2-positive area and proportion of MAFA-positive cells in HFD-fed *Pax4* βiKO mice**

(A) Quantification of GLUT2-positive area normalized to insulin-positive area based on immunofluorescence images using ImageJ. (B) Quantification of MAFA-positive/Insulin-positive cells normalized to total insulin-positive cells in control and *Pax4* βiKO mice. n ≥3 mice per group and more than 100 islets per mouse were analyzed.

**
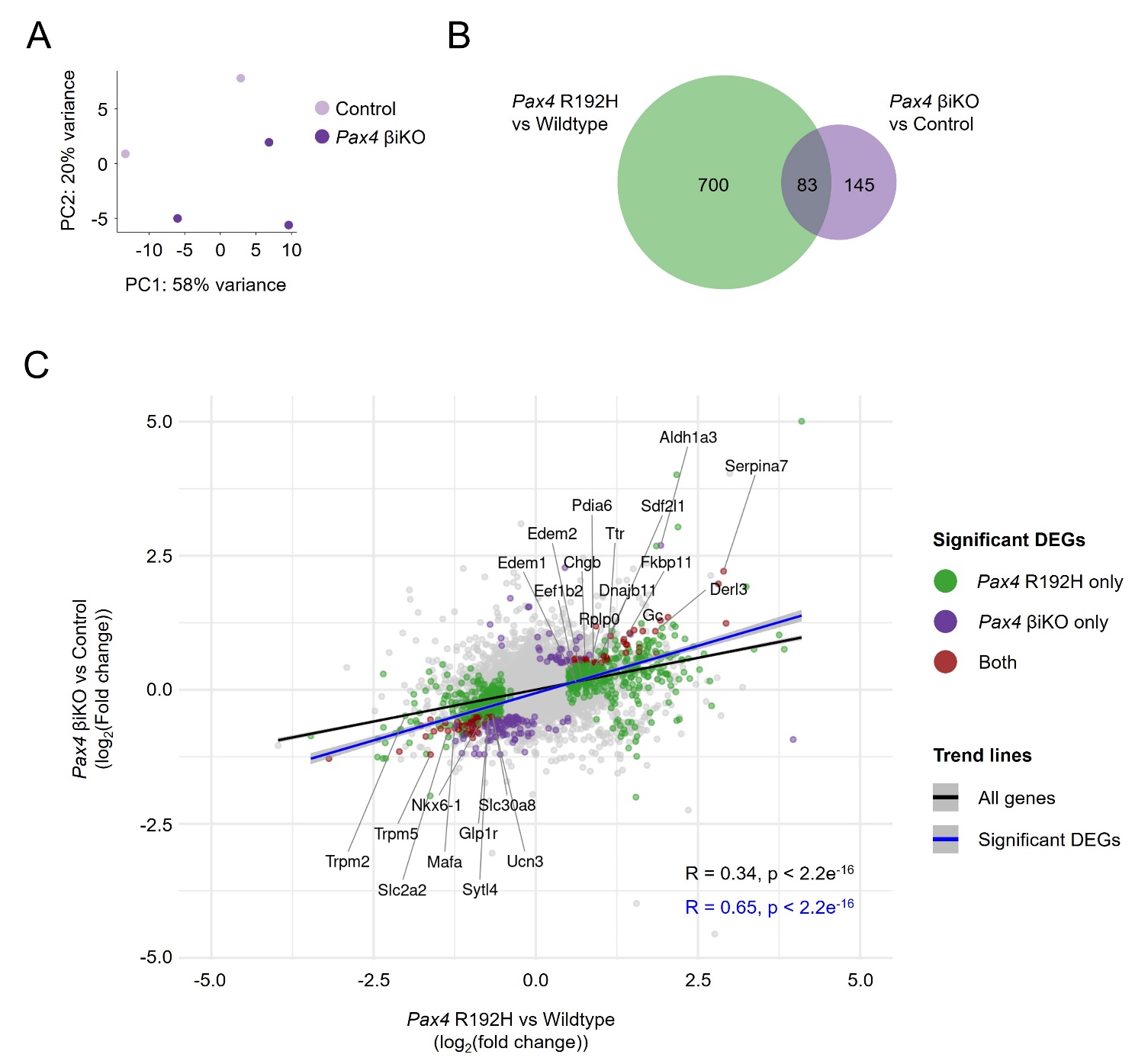
**

**Supplementary Figure 11. Transcriptomic analyses revealed a positive correlation between gene expression changes observed in *Pax4* R192H and *Pax4* βiKO islets*.***(A) Principal component analysis (PCA) of bulk RNA-seq data from control and *Pax4* βiKO islets after 8 weeks of high fat diet (20-week-old). (B) Venn diagram showing the number of differentially expressed genes (DEGs, log_2_(fold change) >0.5, adjusted *P* value <0.05) in islets of *Pax4* R192H mice (compared to wildtype mice, green circle) and *Pax4* βiKO mice (compared to control mice, purple circle) respectively. (C) Scatterplot showing the correlation of log_2_ fold changes of gene expression in islets of *Pax4* R192H mice (compared to wildtype mice) and *Pax4* βiKO mice (compared to control mice). Colored dots indicate significant DEGs (log_2_(fold change) >0.5, adjusted *P* value <0.05) in *Pax4* R192H mice (green), *Pax4* βiKO mice (purple), or both genotypes (red). Trend lines and Pearson correlation coefficient were derived from all genes (black) or significant DEGs (blue).
